# The PI3K/mTOR Pathway Is Targeted by Rare Germline Variants in Patients with Both Melanoma and Renal Cell Carcinoma

**DOI:** 10.3390/cancers13092243

**Published:** 2021-05-07

**Authors:** Jean-Noël Hubert, Voreak Suybeng, Maxime Vallée, Tiffany M. Delhomme, Eve Maubec, Anne Boland, Delphine Bacq, Jean-François Deleuze, Fanélie Jouenne, Paul Brennan, James D. McKay, Marie-Françoise Avril, Brigitte Bressac-de Paillerets, Estelle Chanudet

**Affiliations:** 1 Section of Genetics, International Agency for Research on Cancer (IARC-WHO), 69372 Lyon, France; hubertjn@fellows.iarc.fr (J.-N.H.); maxime.vallee@chu-lyon.fr (M.V.); tiffany.delhomme@irbbarcelona.org (T.M.D.); BrennanP@iarc.fr (P.B.); mckayj@iarc.fr (J.D.M.); 2 Gustave Roussy, Département de Biopathologie, 94805 Villejuif, France; voreak.suybeng@curie.fr (V.S.); fanelie.jouenne@aphp.fr (F.J.); 3 Department of Dermatology, AP-HP, Hôpital Avicenne, University Paris 13, 93000 Bobigny, France; eve.maubec@aphp.fr; 4 UMRS-1124, Campus Paris Saint-Germain-des-Prés, University of Paris, 75006 Paris, France; 5 Centre National de Recherche en Génomique Humaine, Université Paris-Saclay, CEA, 91057 Evry, France; boland@cng.fr (A.B.); bacq@cng.fr (D.B.); deleuze@cng.fr (J.-F.D.); 6 Association Robert Debré pour la Recherche Médicale, 75006 Paris, France; marie-francoise.avril@orange.fr; 7 INSERM U1279, Tumor Cell Dynamics, 94805 Villejuif, France

**Keywords:** melanoma, renal cell carcinoma, genetic susceptibility, rare variants enrichment, WES

## Abstract

**Simple Summary:**

Patients with malignant melanoma have an increased risk of being affected by kidney cancer and vice versa. Lifestyle risk factors contributing to these cancers differ. Instead, our study aims to assess whether common genetic predispositions may be at play. Here we reveal the clinical and germline genetic characteristics of a series of 125 patients diagnosed with both malignant melanoma and renal cell carcinoma (RCC), the most common type of kidney cancer. Clinical testing of known predisposing genes only explains a minority of either or both cancer occurrences. Instead, a wide exploration of all coding genes identified 13 novel susceptibility candidates more prone to rare deleterious germline mutations than expected in cancer-free controls, and converging to a common signaling pathway. This research highlights methods to better characterize cancer (co-)heritability. It also provides a basis to better understand and diagnose melanoma and RCC, which is essential for adequate clinical management.

**Abstract:**

**Background**: Malignant melanoma and RCC have different embryonic origins, no common lifestyle risk factors but intriguingly share biological properties such as immune regulation and radioresistance. An excess risk of malignant melanoma is observed in RCC patients and vice versa. This bidirectional association is poorly understood, and hypothetic genetic co-susceptibility remains largely unexplored. **Results:** We hereby provide a clinical and genetic description of a series of 125 cases affected by both malignant melanoma and RCC. Clinical germline mutation testing identified a pathogenic variant in a melanoma and/or RCC predisposing gene in 17/125 cases (13.6%). This included mutually exclusive variants in *MITF* (p.E318K locus, N = 9 cases), *BAP1* (N = 3), *CDKN2A* (N = 2), *FLCN* (N = 2), and *PTEN* (N = 1). A subset of 46 early-onset cases, without underlying germline variation, was whole-exome sequenced. In this series, thirteen genes were significantly enriched in mostly exclusive rare variants predicted to be deleterious, compared to 19,751 controls of similar ancestry. The observed variation mainly consisted of novel or low-frequency variants (<0.01%) within genes displaying strong evolutionary mutational constraints along the PI3K/mTOR pathway, including *PIK3CD*, *NFRKB*, *EP300*, *MTOR*, and related epigenetic modifier *SETD2*. The screening of independently processed germline exomes from The Cancer Genome Atlas confirmed an association with melanoma and RCC but not with cancers of established differing etiology such as lung cancers. **Conclusions:** Our study highlights that an exome-wide case-control enrichment approach may better characterize the rare variant-based missing heritability of multiple primary cancers. In our series, the co-occurrence of malignant melanoma and RCC was associated with germline variation in the PI3K/mTOR signaling cascade, with potential relevance for early diagnostic and clinical management.

## 1. Introduction

Malignant melanoma and renal cell carcinoma (RCC) are the fifth and seventh most common cancers expected to be diagnosed in 2019 in the United States, accounting respectively for 5% and 4% of all cases [1] and responsible for over 235,000 deaths worldwide in 2018 [2]. The vast majority of malignant melanomas arise from skin, while less than 10% are of ocular, mucosal, or undetermined primary origin [3]. Co-occurrence of both a cutaneous malignant melanoma (CMM) and an RCC represents 0.5% of CMM cases and 1% of RCC cases [4]. We previously described a set of 42 French cases with co-occurrence of both cancers [4]. Despite different embryonic origins, CMM and RCC share biological properties such as immune regulation, radioresistance, as well as patterns of response to immunotherapies [5,6]. Several epidemiological studies consistently reported an increased incidence of melanoma after RCC and vice versa, based on standardized incidence ratios (SIR) of second cancers calculated in different countries, including Italy, the USA, Germany, and Norway [5]. Based on the US population registry, melanoma patients had a 34% increased incidence of RCC, whereas RCC patients had a 45% increased incidence of melanoma [5]. The reasons underlying the bidirectional association between CMM and RCC are not yet elucidated. Non-random causes of multiple occurrences of primary cancer include common environmental/lifestyle factors and/or shared genetic etiology.

Main environmental and host risk factors for CMM include ultraviolet (UV) light exposure, history of sunburn in childhood or adolescence, number or type of melanocytic nevi, and pigmentation [7]. Confirmed risk factors for RCC include tobacco smoking, excess body weight, history of hypertension, and chronic kidney disease [8]. To date, there is no established risk factor common to both melanoma and RCC, although the role of obesity in melanoma warrants further investigations following inconsistent reports in more recent years [9,10].

Common genetic predisposition only explains a minority of malignant melanoma or RCC. Risk loci identified by genome-wide association studies (GWAS) account for about 10% of RCC risk and 19% (USA) to 30% (Australia) of melanoma risk, respectively [11,12]. Among the common loci for melanoma susceptibility, the melanocortin 1 receptor *MC1R* is an outstanding gene. It encodes a transmembrane receptor regulating melanin through the control of melanocyte-inducing transcription factor (*MITF*) expression and activity [13,14]. *MC1R* is a highly polymorphic gene within Caucasian populations as an evolutionary consequence of the migration of ancestral populations to an environment with reduced UV light exposure. These polymorphisms functionally impact various receptor functions, modulating skin photoprotective pigments eumelanin/pheomelanin ratio [14]. Both epidemiological and biochemistry studies documented a carcinogenic role of pheomelanins produced by functionally impaired receptor encoded by some *MC1R* allelic variants, considered as disruptive [15]: suspected underlying mechanisms included increased oxidative stress, inflammation, and immunomodulation [13], resulting in low to moderate melanoma risk [16].

A substantial component of the missing genetic susceptibility may come from rarer variants not addressed by GWAS [17]. In melanoma-prone families, predisposing genes target the cell cycle (*CDKN2A*, *CDK4*) or telomere regulation (*ACD*, *POT1*, *TERF2IP, TERT*) [18] whereas RCC predisposing genes target mainly metabolism, in particular, the Akt/HIF pathway (*FH*, *FLCN*, *MET*, *PTEN*, *SDHs*, *TSC1*, *TSC2*, and *VHL*), and epigenome regulation (*PBRM1* and *BAP1*). Inherited mutations in two genes, *MITF* and *BAP1*, predispose to both CMM and RCC. *MITF* encodes a transcription factor whose M-isoform specifically expressed in melanocytes, coordinates a wide range of biological processes such as cell survival, differentiation, proliferation, invasion, senescence, metabolism, and DNA damage repair [19]. Interestingly, isoform *Mitfa* (widely-expressed) deficiency does not visibly alter mice pigmentation in skin and eye, although it results in reduced nephron number, whereas overexpression of *Mitfa* leads to a substantial increase of nephron number and bigger kidneys [20]. We previously reported a hotspot mutation in *MITF*, p.(E318K), significantly more frequent in melanoma and/or RCC genetically-enriched cases than in controls [21]. The second gene, *BAP1*, has been shown to predispose to various cancers of different embryonic origins, among which the core tumoral spectrum is composed of cutaneous and ocular melanoma, RCC, and mesothelioma [22]. *BAP1* encodes a deubiquitinase regulating a number of processes, including DNA damage repair, cell cycle control, chromatin modification, programmed cell death, and immune responses [23].

Physiologically, both the epidermis (where melanocytes are located) and the inner renal medulla are hypoxic tissues [24,25]. *MITF^E318K^* germline hotspot mutation was shown to impair MITF SUMOylation, to increase the affinity for the hypoxia-induced *HIF1A* promoter, and to enhance migration, invasion, and clonogenicity of melanoma and renal cancer cells [21]. Various environmental stresses, including hypoxia and reactive oxygen species (ROS), were previously shown to induce global protein SUMOylation [26]. In this context, *MITF^E318K^* could impair the adaptation of cells to stress and initiate tumor formation. Among the genes differentially regulated between Mitf^WT^ and Mitf^E318K^ in a mouse model, *CDKN2B* [27] was previously described as an RCC predisposing gene [28]. Besides, *BAP1* deubiquitinase activity is associated with intra-cellular ROS homeostasis and sensitivity to oxidative stress [29]. Altogether, both etiology and biology suggest that malignant melanoma and RCC may share molecular pathogenic pathways, possibly related to oxidative stress cellular responses.

The first aim of the current study is to update the clinical and genetic description of an extended set of 125 patients affected by both melanoma and RCC. The second is to identify potential new candidate co-susceptibility genes through an agnostic approach, performing whole-exome sequencing (WES) on a subset of 46 early-onset patients and testing for gene-based enrichment in rare deleterious variants against large series of external controls.

## 2. Materials and Methods

### 2.1. Recruitment of Patients and Data Collection

A total of 125 cases with a confirmed diagnosis of malignant melanoma and RCC were recruited over a 40 year period (from 1979 to 2018) through French dermatological or oncogenetic clinics, as previously described for the first 42 enrolled cases [4].

### 2.2. Ethic and Consent

The study was approved by the Institutional Review Board (IRB#00001072, CCPPRB Paris Necker and Ethical Committee of Le Kremlin-Bicêtre University Hospital; N°2001-09-05; N°2010-01-09). All subjects gave written informed consent before participation.

### 2.3. Clinical Genetic Testing

All 125 individuals in the cohort had their blood drawn after genetic counseling and diagnostic only or diagnostic and research-informed consent. Germline DNA was extracted using the QIAamp DNA Blood mini kit (QIAGEN, Hilden, Germany), according to the manufacturer’s guidelines [30]. Before 2017, upon clinical indications based on personal and familial cancer history, cases (N = 122) were tested for established melanoma predisposing genes, namely *BAP1*, *CDKN2A*, *CDK4*, *MC1R*, *MITF*, and/or RCC predisposing genes, guided, when applicable, by histological subtypes (*BAP1*, *FH*, *FLCN*, *MITF*, and *VHL*) in a clinical laboratory. Point mutations were screened by Sanger sequencing: for tumor suppressor genes, this included all coding exons ± 25 bp flanking intronic sequences; for the two oncogenes, Sanger sequencing was restricted to the exon with known mutation hotspot (exon 2 for *CDK4* and exon 9 for *MITF*). In addition, genomic rearrangements were searched through quantitative PCR (q-PCR) and multiplex ligation-dependent probe amplification (MPLA), as previously described [30]. One case was sequenced based on phenotypic indications for the familial *PTEN* loss of function germline mutation and was found carrier. From 2017 onwards, three cases were analyzed for melanoma and RCC predisposing genes by multigene panel next-generation sequencing (NGS), including *BAP1*, *CDKN2A*, *CDK4*, *FH*, *FLCN*, *MC1R*, *MET*, *MITF*, *SDHB,* and *VHL* genes; one was a carrier of the *MITF* p.Glu318Lys, the others were wild-type for all genes analyzed. NGS was performed using a library designed to capture all exons ± 50 bp (Capture Agilent SureSelect QXT) then run on a MiSeq Illumina to a minimum depth of 100×. Sequencing data (FastQ files) were generated by MiSeq Analysis software, and subsequently, alignment (GRCh37) and variant calling (including structural variants) were performed with an in-house developed bioinformatics pipeline including BWA alignment [31], haplotype-based GATK variant calling [32], and snpEff annotation [33]. Variants interpretation was performed following the standards and guidelines recommended by the American College of Medical Genetics (ACMG) [34] by board-certified (Agence de la biomédecine, France) clinical molecular geneticists. *MC1R* variants were classified as “R”, moderate melanoma risk (D84E, R142H, R151C, R160W, D294H), and “r”, low melanoma risk (V60L, V92M, I155T, R163Q) [35]. In addition, three variants too rare for melanoma association studies were found, two were associated with red hair color (RHC) in the UK Biobank (T95M and I180fs) [36], and the last one, F196V, was not associated with any functional or genetic data.

### 2.4. Exome Sequencing, Variant Calling, and Filtering

A subset of 46 cases was further investigated by exome sequencing of blood DNA: they were cases among the youngest age of onset, for whom clinical testing did not identify any clinically relevant germline mutation and whose informed consent agreed for anonymous genomic research. Exome captures were performed using a SureSelect Human All Exon V5 kit (Agilent Technologies, Santa Clara, CA, USA). Sequencing was performed on a HiSeq 2000 (Illumina, San Diego, CA, USA), with 100 bp paired-end reads, to achieve minimum on-target coverage of 60 to 70×.

Nextflow-based [37] exome processing pipelines are available through GitHub (https://github.com/IARCbioinfo accessed on 25 January 2021). In brief, sequencing reads were aligned on GRCh38 with BWA v0.7.15 [31], postalT-processed, and duplicate reads were marked with Sambamba v0.6.6 [38]. Variant calling was performed with GATK v4.1.4.1 and strictly followed Best Practices recommendations [32,39] for base quality recalibration, haplotype calling, joint genotyping, variant filtering, and recalibration. Genomic positions with more than 10% missing data and/or heterozygous sites with an alternative allelic fraction of less than 25% were discarded. Sex concordance between clinical and sequencing data was confirmed using PLINK v1.90 [40].

Variants were annotated with ANNOVAR 2020Apr01 version [41]. Variants deviating from expected Hardy–Weinberg proportions were discarded [42]. A variant was considered a rare variant if its allele frequency was equal or inferior to 0.25%, that is, the allele frequency of *MITF* p.E318K hotspot, in any gnomAD v2.1.1 outbred population [43,44]. Variant deleteriousness was assessed using both Combined Annotation Dependent Depletion (CADD v1.4) [45] and ClinVar (version 20200316) [46] databases. Variants with a CADD phred-like rank score ≥ 20, and/or two or more non-conflicting “Pathogenic” or “Likely pathogenic” ClinVar annotations, and/or identified as frameshift indels with mapping quality ≥ 50, were designated as deleterious, that is, likely to impact the function of the encoded protein.

### 2.5. Gene-Based Case-Control Analyses

To identify potential genes enriched in rare deleterious variations in our 46 cases, we implemented the Proxy External Controls Association Test approach (ProxECAT) [47]. The proxECAT-weighted test is tailored to rare variants case-control association analyses using publicly available datasets as control. It uses the synonymous variation information to adjust for differences in data processing. Our external control set consisted of gnomAD European samples assigned to a north-western sub-continental ancestry and not ascertained for having cancer in a cancer study (N = 19,751). In brief, VCF files publicly distributed along with gnomAD v2.1 release were converted to GRCh38 using LiftoverVcf from Picard Toolkit v2.19 (http://broadinstitute.github.io/picard accessed on 25 January 2021), and annotated similarly to our case series. Likelihood-ratio test *p*-values were adjusted using the conservative genomic control factor approach to take into account population stratification [48]. The corresponding *q*-values were computed using the Benjamini–Hochberg procedure to control the false-discovery rate [49].

### 2.6. Validation of Candidate Genes and Variants

Two complementary series of controls were used to evaluate our candidates further. We first check our variants’ loci in an internal control series, consisting of exome samples (N = 288) of Eastern European ancestries, collected as non-cancer controls as part of our previous lung cancer susceptibility study [50], and processed similarly to internal cases. Candidate variants were then searched within a French reference panel available through the French Exome Project (FrEx) database (N = 574) to check for potential population-specific recurrent variation that would be unlikely to cause the observed phenotype (http://lysine.univ-brest.fr/FrExAC, accessed on 25 January 2021) [51].

Variants driving the enrichment of candidate genes identified by the ProxECAT gene-based enrichment test were manually inspected using IGV genome browser v2.5.3 [52] and curated using complementary annotations, including updated region-, gene-, and variant-based annotations from Ensembl release 102 and gnomAD v3.1. Association between genetic variation and clinical parameters, including the age of cancer onset, personal history of cancer, familial history of melanoma or RCC, and histological subtypes, was assessed using the Fisher exact test for categorical variables and the Mann–Whitney U test for continuous variables.

The biological relevance of our candidate genes was evaluated through a literature-based search for a link with disease susceptibility and/or cancer development. A functional pathway enrichment analysis was also performed with g:Profiler (version e102_eg49_p15_7a9b4d6) [53] using the Kyoto encyclopedia of genes and genomes (KEGG) and WikiPathways as biological pathway sources. Additionally, we screened similar cancer series from The Cancer Genome Atlas (TCGA), namely skin cutaneous melanoma (SKCM, N = 470), kidney renal clear cell (KIRC, N = 344), and kidney renal papillary cell (KIRP, N = 289). To assess potential enrichment in those series compared to other cancer types of established differing etiology, we extended the screening to two additional TCGA series of similar size and a similar proportion of overall deleterious variants carriers [54], i.e., lung adenocarcinoma (LUAD, N = 540) and lung squamous cell carcinoma (LUSC, N = 514). In brief, TCGA exomes (TCGA access #2731) were acquired from the Institute for Systems Biology Cancer Genome Cloud (release 1.1, https://isb-cgc.appspot.com accessed on 25 January 2021). Germline variant calling was performed in-house using Platypus (https://github.com/IARCbioinfo/platypus-nf accessed on 25 January 2021) [55]. Rare deleterious variants affecting our 13 candidate genes in any of those series were reported, together with loci reported as familial cancer susceptibility variant in the literature, irrespective of the type of familial cancer affected.

## 3. Results

### 3.1. Overview of Clinical Sequencing Results

The demographic and histological characteristics of the 125 cases with both malignant melanoma and RCC are detailed in Table 1. Upon clinical indication based on personal and familial cancer history and, when applicable, RCC histological subtypes, cases were tested for established melanoma and/or RCC predisposing genes. In addition, all cases were tested for germline *MITF* mutations as part of a translational research work and nine cases carried the germline missense substitution p.E318K: this mutation was predominantly observed in men (8/9), cases were frequently affected by more than one CMM (4/9), and the associated RCC subtypes were diverse (Table 2). Three carriers of *BAP1* pathogenic mutations were detected out of ten individuals tested. Sixty-eight individuals were tested for both *CDKN2A* and *CDK4* mutations, among whom only two were carriers of a *CDKN2A* pathogenic mutation. Two carriers of a pathogenic *FLCN* mutation were detected out of the ten tested. Both patients showed clinical signs suggestive of Birt–Hogg–Dubé syndrome, namely fibrofolliculoma and leiomyosarcoma, respectively. One individual was tested for the familial pathogenic germline *PTEN* mutation and was found carrier, as well as her acral melanoma affected daughter. No carriers of pathogenic mutation were found out of 47 tested for *VHL*, and 57 for *FH*. In total, 17/125 (13.6%) were carriers of a pathogenic germline mutation (Table 2). Among these 17 cases, 12 (70%) carried, in addition, at least one *MC1R* variant.

### 3.2. WES Confirmed Infrequent Pathogenic Variants in Melanoma and/or RCC Risk Genes

A subset of 46 cases, among the youngest age of onset and wild type upon diagnostic testing indication, was selected for further exploration by exome sequencing. Variant calling yielded a total of 3 × 10^5^ variants evenly distributed across samples, with a median number of 89,593 variants per case. This included an average of 130 rare (AF ≤ 0.25%) variants hereto defined as ‘deleterious’ (CADD ≥ 20, and/or congruent ClinVar annotations of pathogenicity, and/or high-quality frameshift).

We first looked at an extended list of high-risk genes predisposing to melanoma (*ACD, CDKN2A, CDK4, POT1, TERF2IP,* and *TERT*) [56] or RCC (*CDKN2B, FH, FLCN, MET, PBRM1, PTEN, SDHs, TSCs,* and *VHL*) or both (*BAP1* and *MITF*) [57]. Exome sequencing confirmed the absence of any rare deleterious variation in genes included in the clinical genetic testing, with one exception. A stop-gain variant (p.W306*; rs142934950) in the shorter isoform (isoform 2) of *FLCN* (NM_144606.6) was observed in two cases (Supplementary Table S1). Manual inspection revealed that the variant was located in the intronic sequence of the reference transcript used in clinical practice (RefSeq NM_144997). No conclusion could be drawn about pathogenicity as the function of *FLCN* isoform 2 has not yet been elucidated [58]. Further, a single unpublished and very rare (AF ≤ 0.01%) missense variant (rs1303562362) was observed in *ACD* (p.L511R, CADD of 26.1), affecting a highly conserved residue located in the C-terminal *TINF2* binding domain [485–544] [59]. *ACD* encodes a protein of the shelterin complex, which protects chromosomal ends and is required to inhibit the elongation of chromosome ends in somatic cells. *ACD* loss of function (LOF) mutations predispose to melanoma and a broader spectrum of cancers [59]. Despite the fact that no RCCs were reported in *ACD* carriers to date [59,60,61], a meta-analysis suggested that individuals with an inherited predisposition to longer telomere length are at increased risk of developing renal cell carcinoma [62]. Two other cases respectively harbored a novel 14-base-pair deletion (c.2228_2240del p.(Q743fs)) and a rare missense VUS (rs1588304158) in *TSC1*. *TSC1* is a tumor suppressor involved in the control of mTOR activation [63]. Germline heterozygous mutations in *TSC1* are known to be responsible for hamartoma syndromes, including tuberous sclerosis (TS) that confer increased susceptibility to renal cancer [64]. Of note, the *TSC1* variants observed in our series were located in exons 17 and 18, encoding part of the tuberin-binding region regularly targeted in TS [65].

### 3.3. Gene-Based Case-Control Analysis Identified 13 Candidate Susceptibility Genes

**Figure 1:**
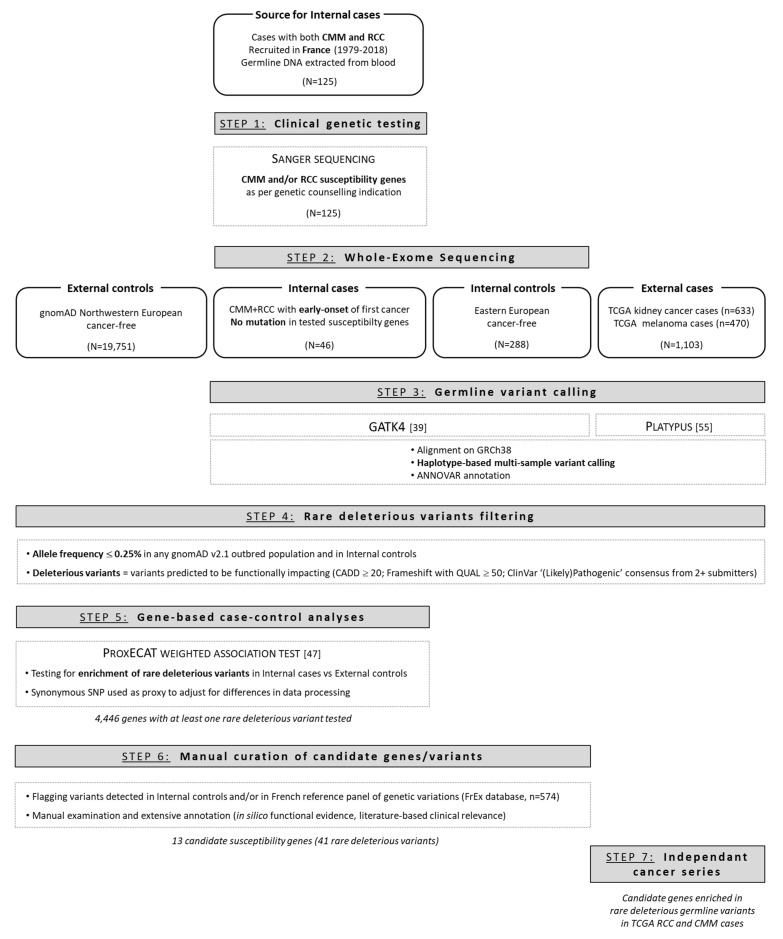
Flow chart of our candidate susceptibility genes discovery approach.

To elucidate further malignant melanoma and RCC potential shared genetic susceptibility, we applied an agnostic approach that consisted in assessing gene-based enrichment in rare deleterious exonic variants in our series compared to large series of external non-cancer controls from similar ancestry (Figure 1). The control set encompassed gnomAD non-cancer individuals from north-western European ancestry (N = 19,751). We used the ProxECAT test [47], an allele-frequency-based association test allowing us to make the most of publicly available control datasets, while controlling for differences in internal versus external data processing via synonymous variants.

A total of 4446 genes that displayed at least one variant matching our rarity and deleteriousness criteria were tested. Given the distribution of gene-based *q*-values (Supplementary Table S2 and Supplemental Information), further analyses were restricted to genes displaying a *q*-value of 0.2 or less. A total of 13 genes displayed a significant enrichment in rare deleterious variants in cases compared to controls, namely *PIK3CD*, *MTOR*, *RAE1*, *ZBTB21*, *ESAM*, *TMEM192*, *CLTCL1*, *NFRKB*, *EP300*, *MTSS2*, *SETD2*, *SMC2,* and *EBF4* (Table S3, Supplemental Information). Most candidate genes showed strong evolutionary mutational constraint, arguing against the random accumulation of functionally impacting mutations (Table 3). Altogether, they comprised 41 distinct rare deleterious variants (Table 4). Twenty-five of them (61%) were novel or very rare variants (MAF < 0.01%). Overall, 33 of our 46 cases (72%) showed at least one mutation in at least one candidate gene (Table S1). Combined with the *ACD*, *TSC1,* and *FLCN* mutations uncovered in the previous search focusing on known susceptibility genes to CMM or RCC, potential candidate(s) were identified in 34 of 46 cases (74%; Table S1). This was not significantly different in cases with a family history of CMM and/or RCC (four of five cases with at least one affected first-degree relative, i.e., 80% of the cases with a positive family history) versus sporadic cases (30/41 = 73%). The majority of the mutations were exclusive: a single hit in a unique gene was observed in 29 of 34 cases, i.e., 85% of the mutated cases. While the concordance rate between the two methods of *MC1R* variants identification (exome sequencing versus clinical sequencing) was 100%, there were no differences in the *MC1R* status (presence of disruptive and/or non-disruptive variants versus absence of variant) according to the above mutational status. In our series, there was a trend for *SETD2* and *CLTCL1* mutations to be associated with cases with a personal history of solid tumors (six of the eight cases with *CLTCL1* or *SETD2* variants had at least one other solid tumor vs. 11/38 cases without a variant in *CLTCL1* nor *SETD2*; *p* = 0.04; Table S1).

Further assessment of candidate genes was based on the use of two complementary series of controls: an internal control set accounting for potential calling bias (288 non-cancer cases from European ancestries with identical data processing) and an external control set from the exact same ancestry to flag potential population-specific polymorphisms (574 non-disease cases of French origin). No candidate variant could be detected from the internal control set, which suggested that rare variations observed in cases were unlikely to result from technical artifacts. Six variants from five candidate genes (*ESAM*, *SETD2*, *MTSS2*, *SMC2*, and *EBF4*) were found in the French external control set, albeit at very low frequencies consistent with those observed in other populations as per current gnomAD annotations. This observation favored the hypothesis of shared (very) rare variants similarly segregating in various populations rather than recently acquired population-specific polymorphisms.

### 3.4. Pathway Level Analyses Highlighted the Central Role of PI3K/Akt and Its Downstream mTOR/HIF Axis

**Figure 2:**
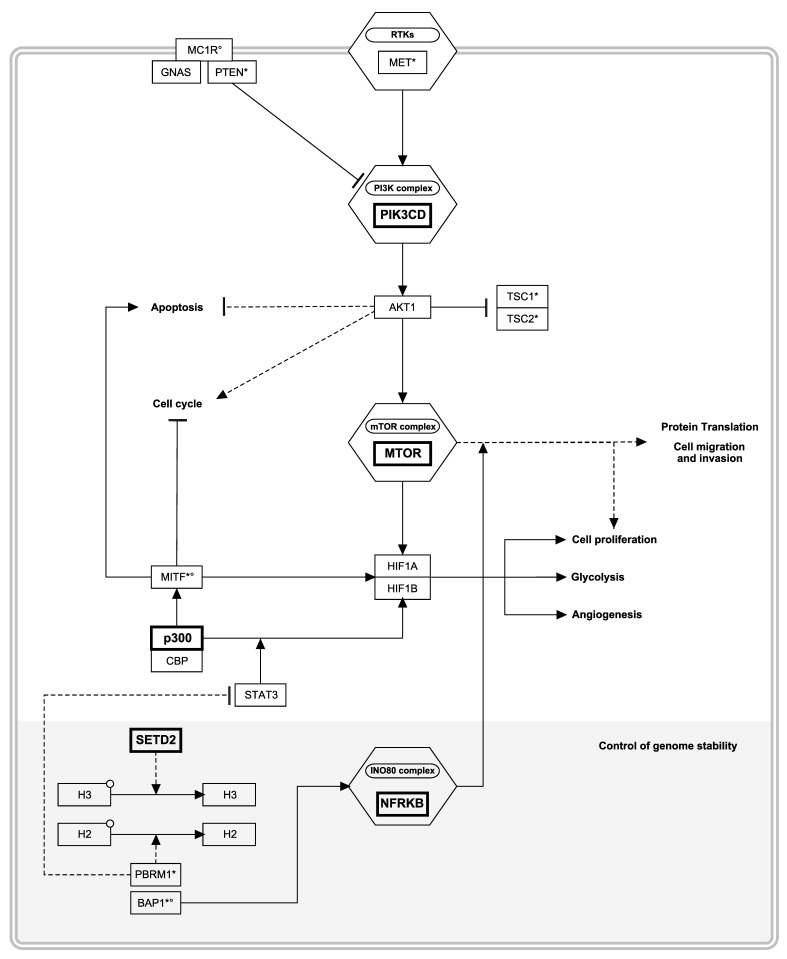
Overview of the PI3K/Akt/mTOR signaling pathway. Newly identified candidate genes are highlighted in bold. ° Established CMM-predisposing genes. * Established RCC-predisposing genes. RTKs: Receptor tyrosine kinases; H2, H3: histones. This figure was built from the “Pathways in clear cell renal cell carcinoma” pathway [68] hosted on WikiPathways [69].

The most significant enrichment in rare deleterious germline variations was found in *PI3KCD* and *MTOR* (FDR *q*-value ≤ 0.05), two genes that belong to the PIKK protein kinase family, acting along the PI3K/Akt/mTOR axis. Two additional genes among the 13 candidates had documented functions in the same pathway: *NRFKB* and *EP300* (Figure 2). This pathway is closely related to epigenetic modifiers in charge of maintaining genome integrity, such as *SETD2* [66]. Overall, a large proportion of mutated cases (14/34 = 41%) had a novel or rare variant affecting at least one of the four PI3K/Akt genes (N = 12) identified from our agnostic approach, or the RCC susceptibility gene *TSC1* (N = 2) that belongs to the same axis (Figure 2). Rare deleterious germline variations within the PI3K/Akt pathway (*PIK3CD*, *MTOR*, *EP300*, *NFRKB*, and *TSC1*) were consistently mutually exclusive. Of note, PI3K/Akt affected cases tend to have a younger onset of both melanoma (median age of onset at 46 years old for cases with a rare deleterious variant in the PI3K/Akt pathway versus 53.5 years old without, *p* = 0.15) and RCC (48.5 vs. 55.5 years old, *p* = 0.07). No significant associations with RCC or melanoma subtypes or *MC1R* status or with a personal history of cancer were observed.

Pathway enrichment analysis of our 13 candidate genes confirmed direct connections with signaling pathways dysregulated in cancer (Table 5), including signaling cascades downstream of tyrosine kinase receptors notably involved in pancreatic and renal cancer, such as HIF-1 and JAK-STAT signaling pathways. Over-representation was driven by *PIK3CD*, *MTOR*, *EP300,* and *SETD2*, all known to be involved in RCC development. Although the related gene set size did not allow to reach significance, the melanoma canonical pathway included two of them, namely *PIK3CD* and *EP300* [69,70].

### 3.5. Relevance of Our Candidate Susceptibility Genes in Malignant Melanoma and RCC

The biological relevance of the 13 novel candidate genes in cancer development was assessed through a dedicated literature search (File S1) combined with a representation of the structural/functional organization of the affected proteins (File S2). In brief, the protein functions associated with our candidate genes mainly pointed to downstream PI3K signaling and genome integrity, with frequent direct implications in CMM and RCC development. A few candidate genes, such as *ZBTB21*, *MTSS2,* and *EBF4,* still have elusive functional mechanisms, while belonging to families of genes with suggested roles in cancer susceptibility. Of note, the *NFRKB* variant rs200192480 (c.C2113T, p.P705S) was reported as segregating in one family with five members affected by papillary thyroid cancer [67].

In parallel, we checked the occurrence of the 41 identified variants spanning the 13 novel candidate genes in cancer cases from the TCGA series of CMM (SKCM, N = 470) and RCC (KIRC and KIRP, N = 633) [71]. The processing pipeline, including the germline caller, used for the TCGA series was different from that used in our cases: concordant calls are thus unlikely to be technical artefact. In total, 7 of 41 variants (17%) were identified in 16 cases, including 8 SKCM and 8 RCC cases (Table 4). *MTOR* variant rs142403193, which is located in the PI-kinase FAT domain, was reported four times, affecting two cases in our discovery set and two SKCM cases. As *EP300* variant rs201480900 and *SETD2* variant rs114719990, it was found at higher frequency in TCGA SKCM series compared to any gnomAD populations. In TCGA series, as in ours, *SETD2* variation spanned melanoma and different kidney cancer subtypes, which is in line with the documented broad role of *SETD2* in cancer [72,73]. Besides, our set of variants was significantly enriched (*p* = 0.04) in TCGA relevant series (SKCM, KIRC, and KIRP) compared to TCGA series of known differing etiology that were lung adenocarcinoma (LUAD, N = 540) and lung squamous cell (LUSC, N = 514), while there were no differences in accumulating rare deleterious variants overall. Supplementary Table S3 lists all rare deleterious variants affecting one of the 13 genes in at least one individual from TCGA CMM and/or RCC.

Altogether, our investigations suggested that novel candidate genes may contribute to explain the inherited genetic basis of malignant melanoma and/or RCC.

## 4. Discussion

We hereby provided a clinical and genetic description of a series of 125 cases affected by both malignant melanoma and RCC.

In line with our initial observations [4], only a minority of the cases (12/125; 9.6%) could be explained by a clinically validated pathogenic variant in one of the two known genes predisposing to both melanoma and RCC, namely *MITF* (N = 9) and *BAP1* (N = 3). In total, 9 out of 125 patients (7.2%) notably displayed the *MITF* p.E318K germline mutation [21]. Although the role of this variant in melanoma predisposition has been confirmed by numerous reports [74,75,76], its role in RCC susceptibility has not been fully recognized. Two RCC case controls studies were negative [77,78]. However, the first study also failed to find an association with melanoma [77] and the second was performed on FFPE tissue [78]. RCC frequent somatic 3p losses, related to three major RCC tumor suppressor genes, namely *VHL* (located at 3p25.3), *BAP1,* and *PBRM1* (3p21.1), could have masked germline *MITF* p.E318K alleles (3p13). Two recent case reports identified this mutation in RCC-only cases, including in a 43 year old African American patient affected with bilateral and multifocal type 1 papillary RCC (PRCCI) whose father developed, at 56 years old, a PRCCI with clear cell features [79,80]. Of note, downstream targets of *MITF* were deregulated in the PRCCI tumors, documenting *in vivo* a role of *MITF* p.E318K variant in renal oncogenesis [80]. Taken together, these results and ours support *MITF* p.E318K as a risk allele for the development of RCC.

Our three patients carriers of a *BAP1* pathogenic mutation that belonged to typical *BAP1*-tumor predisposition syndrome (TPDS) families with established increased co-susceptibility to RCC and melanoma [81]. An additional five cases displayed clinically pathogenic variant in three genes predisposing to either melanoma or RCC, namely *FLCN* (N = 2), *CDKN2A* (N = 2), and *PTEN* (N = 1). Few case reports raised the question of a possible role of *FLCN* through the mTOR pathway in CMM susceptibility [82,83], deserving larger studies. Up to date, there is no clear involvement of *CDKN2A* in RCC susceptibility, while somatic alterations of *CDKN2A* are relatively frequent in RCC tumors [84]. Germline mutations in *PTEN* have been associated with an increased risk of a variety of cancer, recently extended to RCC, and to a lesser extent, CMM [85].

To complement our clinical analyses, we implemented an exome-wide agnostic approach in search of rare variants predicted to be functionally impacting and specifically enriched in a subset of 46 unexplained cases among the earliest age of first cancer onset. A large proportion of the whole exome-sequenced cases (15/46) harbor a single rare or novel deleterious germline variant in a gene from the PI3K/Akt signaling cascade: newly identified candidate susceptibility genes included *PIK3CD, MTOR, EP300,* and *NFRKB*. The PI3K/Akt pathway is among the most frequently somatically mutated in cancer [86]. Broad activation of the PI3K/Akt signaling is common in both CMM and RCC, with key genes such as *MTOR, PTEN, BAP1, PIK3CA* frequently harboring somatic mutations, also largely mutually exclusive [86,87,88].

In our series, the PI3K/mTOR axis was targeted in sporadic cases as well as in cases with a positive family history of melanoma. Germline loss of function in regulators of the PI3K/Akt cascade is associated with a range of overgrowth and cancer-predisposing syndromes [89]. Established increased risk of renal cancer and/or melanoma is observed with *BAP1* mutations responsible for *BAP1*-TPDS, *PTEN-AKT1/2-PIK3CA* induced *PTEN*-opathies [90], as well as mTOR signaling syndromes such as *TSC1/2* tuberous sclerosis complex [91]. Extending clinical testing of familial melanoma cases of unknown etiology to additional targets from the PI3K/Akt family might support clinical management further, especially in the context of PI3K/Akt/mTOR inhibitors being actively considered as therapeutic approaches [92,93].

Downstream of the PI3K/Akt cascade lies the hypoxia-inducible transcriptional factor HIF, specifically highlighted by our pathway enrichment analyses. HIF regulation is targeted in hereditary kidney cancer, and constitutive HIF activation, induced by VHL inactivation, is the major molecular signature of RCC [94]. Based on the initial discovery of *MITF* p.E318K mutation, confirmed as a recurrent germline mutation in our series, we previously proposed that *MITF* p.E318K could impair the adaptation of cells to stress and initiate both melanoma and/or RCC tumor formation [21]. Kim and colleagues recently demonstrated that melanoma growth is driven by direct control of *MITF* by the evolutionary conserved master transcriptional coactivator *EP300* [95]. We observed novel or rare deleterious *EP300* mutations in three of our wild-type MITF cases. Heterozygous germline *EP300* mutations were first described in Rubinstein–Taybi syndrome (RBTS), a congenital neurodevelopmental disorder associated with renal development abnormalities and an increased risk of chronic kidney diseases [96]. The histone acetyltransferase encoded by *EP300* is known to initiate hypoxic responses by coupling with the HIF alpha subunit [97], thus enabling the induction of a range of hypoxia-responsive genes critical for tumor angiogenesis, invasion, and immune escape [98,99]. Detailed investigations of the role of the hypoxic tumor microenvironment in melanoma are warranted.

While cancer susceptibility may not be limited to coding regions of the genome, our exome-wide agnostic approach has the advantage to allow the identification of novel susceptibility genes, unlike the majority of cancer susceptibility studies so far limited to a candidate-based approach [100,101]. Overlap among exome/genome-wide studies focusing solely on melanoma or RCC predisposing genes remains limited [101], mostly due to differences in candidate genes/variants prioritization strategies. Nevertheless, most of the new candidates uncovered here are highly conserved genes intolerant to loss of function mutations. Some are directly related to previously identified candidate susceptibility genes, such as EBF family member 4 that shares multiple functional domains with EBF family member 3 suggested to predispose to hereditary melanoma [102]. Other candidates harbored identical rare variants within the TCGA kidney and/or melanoma series, such as *MTOR*.

The genetic heterogeneity observed in our series is not surprising in the context of susceptibility to complex diseases such as cancer [79,103,104]. The common biological pathways highlighted by our results suggest possible shared co-susceptibility to CMM and RCC and possibly other cancers, like that underlaid by the *BAP1* gene. Indeed, candidate genes involved in maintaining genome stability, such as *RAE1*, *SETD2,* and *CLTCL1* [105,106,107], are attractive candidates for broad cancer susceptibility. Germline mutations in genome integrity keepers have long been recognized as a direct cause of increased cancer risk, as extensively demonstrated in breast cancer [108] as well as cancer-predisposing syndromes [109]. This is in line with our observation of an increased personal history of cancer, including cancer beyond CMM and RCC, in cases with *CLTCL1* or *SETD2* mutations. Whether some of the susceptibility genes uncovered in our study may or may not be solely related to CMM risk or RCC risk, as the extent of that genetic component in their overall susceptibility, is yet to be documented by dedicated epidemiological studies, that could also investigate potential gene-environment interactions.

As expected in Caucasian populations, the *MC1R* gene implicated in skin pigmentation was highly polymorphic in our series [110]. Over two-thirds of the patients, irrespective of their status regarding susceptibility genes/candidate genes variations carried at least one *MC1R* variant. In melanocytes, UVB exposure triggers the interaction of *PTEN* with wild-type *MC1R*, but not with functionally deficient variants, leading to Akt inactivation [111]. Actual knowledge about *MC1R* mainly comes from skin melanocytes, pigmentation, and associated pathologies studies, while *MC1R* is also expressed in the kidney where its main natural ligand is the adrenocorticotropic hormone; downstream effects include anti-inflammation and immunomodulation to protect kidney cells from various stress [112]. *MC1R* also interacts with the signal transducer *GNAS* [113], recently suggested to be tumor-promoting in RCC [114]. Given the similarity of pathways involved in melanoma and RCC biology, a possible role of *MC1R* variants in renal cell physiology and RCC deserves additional investigations.

The main limitation of this study is the absence of functional validation in cell lines and animal models that could ascertain the biological consequences of the observed rare genetic variations before considering any new target in clinical testing panels. This is of particular relevance in the context of the broad variability of the human germline landscape, including context-dependent mutation rate differences [115]. However, our investigations compiled evidence in favor of bona fide susceptibility genes. First, our discovery phase included a very large set of ancestry-matched non-cancer individuals to control for germline variation load and tolerance to functionally impacting variations. Second, potential technical artifacts, such as coverage or calling bias, were accounted for from our discovery phase onwards. Beyond state-of-the-art data processing and stringent quality criteria, this included manual inspection of candidate variants and their sequence context in cases as well as in internal non-cancer controls processed similarly, and replication at the variant or gene-level within external series based on different sequencing technologies and processing pipelines (FrEx, TCGA). In the absence of corresponding tumor tissues, further in silico functional assessment included comprehensive annotations with a range of pathogenicity scores and curated information on clinical relevance, gene-wide mutational constraint, as well as somatic alterations. Finally, our study design did not allow the assessment of the potential impact of the identified germline alterations on treatment and outcome, warranting further dedicated investigations.

## 5. Conclusions

Our study highlights that an exome-wide case-control enrichment approach may contribute to better characterize cancer susceptibility grounded on rare variants underexplored to date. Based on our results, germline variations in the PI3K/mTOR signaling cascade are overrepresented in patients diagnosed with both RCC and CMM. Our study pinpoints that both diseases may share molecular pathogenic pathways related to oxidative stress cellular responses, with potential relevance for early detection, diagnosis, and clinical management.

## Figures and Tables

**Table 1:** Characteristics of the 125 patient cases diagnosed with both melanoma and renal cell carcinoma (RCC).

**Demographics**		
No. of patients	125	
No. of male	80	64.0%
No. of female	45	36.0%
Age at 1st melanoma diagnosis	57.3	
Age at 1st RCC diagnosis	58.8	
**Melanoma features**		
Melanoma site		
Cutaneous	158	97.5%
Ocular	1	0.6%
Mucosal	1	0.6%
Unknown	2	1.2%
Histologic subtype for cutaneous melanoma		
Superficial Spreading Melanoma	87	55.1%
Nodular Melanoma	21	13.3%
Lentigo Malignant Melanoma	4	2.5%
Acral Lentiginous Melanoma	2	1.3%
Unclassified	7	4.4%
Unknown	37	23.4%
Year of melanoma diagnosis	from 1984 to 2018	
**RCC features**		
RCC type		
Clear cell	93	72.7%
Papillary	16	12.5%
Chromophobe	8	6.3%
Other	5	3.9%
Unknown	6	4.7%
Year of RCC diagnosis	from 1979 to 2018	

**Table 2:** Characteristics of the 17 patients with pathogenic variants in known melanoma and/or RCC predisposing genes.

Predisposing Gene	Reference Transcript	Nucleotide Change	Amino Acid Change	MC1R Status (Class)	Sex	Age at First Melanoma	No. Melanoma	Melanoma Histological Subtype	Age at First RCC	RCC Histological Subtype	Other Cancers in Proband	Cancers in Family
*MITF*	NM_000248.3	c.952G>A	p.E318K	p.R163Q (r)	Male	33	2	SSM	27	chRCC		Uncle: skin cancer
MITF *	NM_000248.3	c.952G>A	p.E318K	p.V92M (r)	Male	37	1	SSM	55	ccRCC		
MITF *	NM_000248.3	c.952G>A	p.E318K	p.V60L (r) p.R160W (R)	Male	62	1	NM	51	ccRCC		
MITF *	NM_000248.3	c.952G>A	p.E318K	WT	Male	52	1	SSM	52	ccRCC		Mother: breast cancer Maternal uncle: colo-rectal cancer Paternal uncle: leukemia
*MITF*	NM_000248.3	c.952G>A	p.E318K	p.R160W (R) p.D294H (R)	Female	56	2	SSM	59	ccRCC	Basal cell carcinoma	Mother: RCC + lung cancer Sister: basal cell carcinoma
*MITF*	NM_000248.3	c.952G>A	p.E318K	p.R163Q (r)	Male	60	1	SSM	60	chRCC	Thyroid adenocarcinoma (60)	Father: RCC?
MITF *	NM_000248.3	c.952G>A	p.E318K	p.V60L (r)	Male	69	1	NM	69	ccRCC		
MITF *	NM_000248.3	c.952G>A	p.E318K	p.R160W (R)	Male	75	2	NM	70	ccRCC		
*MITF*	NM_000248.3	c.952G>A	p.E318K	p.V92M (r) p.R151C (R)	Male	74	3	SSM	74	pRCC	Basal cell carcinoma	Mother: 2 CMM? Sister: CMM
*BAP1*	NM_004656.3	c.37+1delG	p.?	p.V60L (r) p.R160W (R)	Female	29	6	SSM	49	ccRCC		Father: mesothelioma Sister: OMM Brother: CMM + lung cancer (no tobacco)
*BAP1*	NM_004656.3	c.78-79del	p.V27fs	WT	Male	45	1	NM	53	ccRCC with a sarcomatoid feature		Sister: OMM (53) + lung cancer (53) Nephew: OMM (18) Mother: liver cancer (43) Maternal cousin 1: skin (55) + duodenal cancers (56) Maternal cousin 2: lung cancer (53)
*BAP1*	NM_004656.3	c.1938T>A	p.Y646 *	p.V60L (r)	Female	48	1	SSM	59	ccRCC	Urothelial cancer (59)	Mother and sister 1: CMM Sister 2: meningioma
*CDKN2A*	NM_000077.4	c.146T>G	p.I49S	p.V92M (r) p.R151C (R)	Female	31	1	SMM	36	ccRCC		Mother and sister: CMM
*CDKN2A*	NM_000077.4	c.159G>C	p.M53I	p.V60L (r) p.R151C (R)	Male	46	1	NM	61	ccRCC		Mother and brother: CMM
*FLCN*	NM_144997.6	c.663dupG	p.M222fs	WT	Female	48	1	NM	43	chRCC with oncocytoma components	Leiomyosarcoma	Father: lung cancer Paternal uncle: RCC
*FLCN*	NM_144997.6	c.755dupC	p.C253fs	WT	Male	64	1	SSM	62	ccRCC	Cutaneous fibrofolliculoma	
*PTEN*	NM_000314.6	c.959T>G	p.L320*	WT	Female	55	1	SSM	55	ccRCC		Daughter: ALM (25) with *PTEN*+

* already included in [21]. R: moderate-risk variant in melanoma [35]; r: low-risk variant in melanoma [35]; OMM: oral malignant melanoma; CMM: cutaneous malignant melanoma; ALM: acral lentiginous melanoma.

**Table 3:** Candidate susceptibility genes enriched in rare variants predicted to be deleterious among 46 French cases with CMM and RCC compared to ancestry-matched cancer-free controls.

HGNC Gene Symbol	Gene Description	Gene Length (pb)	LOEUF Mutational Constraint ^a^	Rare ^b^ Deleterious Allele Counts		*p*-Value ^c^	*q*-Value ^d^
				Internal Cases (N = 46)	External Controls (N = 19,751)		
PIK3CD	Phosphatidylinositol-4,5-Bisphosphate 3-Kinase Catalytic Subunit Delta	6333	**0.20**	2	68	2 × 10^−5^	0.04
MTOR	Mechanistic Target of Rapamycin Kinase	12,163	**0.18**	4	252	4 × 10^−5^	0.05
RAE1	Ribonucleic Acid Export 1	5642	**0.19**	2	18	8 × 10^−5^	0.08
ZBTB21	Zinc Finger and BTB Domain Containing 21	8062	**0.25**	3	120	2 × 10^−4^	0.12
ESAM	Endothelial Cell Adhesion Molecule	2920	0.59	2	37	2 × 10^−4^	0.12
TMEM192	Transmembrane Protein 192	10,182	1.29	2	30	3 × 10^−4^	0.13
CLTCL1	Clathrin Heavy Chain Like 1	10,052	0.80	6	438	3 × 10^−4^	0.13
NFRKB	Nuclear Factor Related to KappaB Binding Protein	6335	0.37	3	233	3 × 10^−4^	0.13
EP300	E1A Binding Protein P300	11,692	**0.10**	3	266	4 × 10^−4^	0.15
MTSS2	MTSS I-BAR Domain Containing 2	4986	**0.31**	4	206	4 × 10^−4^	0.15
SETD2	SET Domain Containing 2, Histone Lysine Methyltransferase	10,245	**0.21**	5	505	6 × 10^−4^	0.16
SMC2	Structural Maintenance of Chromosomes 2	6470	**0.23**	4	131	6 × 10^−4^	0.17
EBF4	EBF Family Member 4	3541	0.70	3	72	8 × 10^−4^	0.18

^a^ Continuous gene-level mutational constraint metric (loss-of-function observed/expected upper bound fraction) [44]: low LOEUF scores indicate strong selection against predicted loss-of-function variation in the given protein-coding gene. Scores below 0.35 are indicated in bold. ^b^ Allele frequency ≤ 0.25%, that is MITF p.E318K allele frequency in European population. ^c^ ProxECAT enrichment test weighted statistics [47] using genomic control factor to take into account population stratification [48]. ^d^ Benjamini–Hochberg false-discovery rate [49]; cut-off for statistical significance: *q* < 0.2.

**Table 4:** Set of 41 rare deleterious variants observed in the 13 candidate CMM and/or RCC susceptibility genes identified by gene-based case-control enrichment test.

Chr	Start	End	Ref	Alt	HGNC Gene Symbol	Accession Number	Reference Transcript	Nucleotide Change	Amino acid Change	CADD	AF_Cases	AF_ FrEx	AF_nc_nwe	AF_Popmax	Independent Cancer Series *
1	9715647	9715647	A	G	PIK3CD	.	NM_005026	c.A248G	p.E83G	25	0.01	.	.	.	
1	9715709	9715709	C	T	PIK3CD	.	NM_005026	c.C310T	p.R104C	35	0.01	.	3 × 10^−5^	9 × 10^−6^	
1	11130641	11130641	G	A	MTOR	rs142403193	NM_004958	c.C5501T	p.T1834M	22.8	0.02	.	9 × 10^−4^	7 × 10^−4^	SKCM (2)
1	11238522	11238522	G	A	MTOR	rs751242124	NM_004958	c.C1882T	p.R628C	28.9	0.01	.	.	5 × 10^−5^	
1	11248030	11248030	T	A	MTOR	rs761323069	NM_004958	c.A905T	p.D302V	23.1	0.01	.	.	3 × 10^−4^	
20	57365381	57365381	C	T	RAE1	rs755561880	NM_003610	c.C314T	p.S105L	31	0.01	.	.	6 × 10^−5^	
20	57365432	57365432	A	G	RAE1	.	NM_003610	c.A365G	p.Q122R	20.6	0.01	.	.	.	
21	41991340	41991340	G	A	ZBTB21	rs368359632	NM_001098402	c.C2756T	p.T919M	25.3	0.01	.	.	7 × 10^−4^	
21	41992058	41992058	G	A	ZBTB21	rs371004245	NM_001098402	c.C2038T	p.R680C	26.5	0.01	.	1 × 10^−4^	1 × 10^−4^	
21	41992762	41992762	C	T	ZBTB21	.	NM_001098402	c.G1334A	p.R445H	30	0.01	.	5 × 10^−5^	4 × 10^−5^	
11	124753942	124753942	G	A	ESAM	rs760488150	NM_138961	c.C877T	p.R293W	34	0.01	.	5 × 10^−5^	3 × 10^−5^	
11	124754658	124754658	G	A	ESAM	rs200924772	NM_138961	c.C713T	p.T238M	33	0.01	9 × 10^−4^	8 × 10^−5^	2 × 10^−4^	
4	165103020	165103020	T	G	TMEM192	.	NM_001100389	c.A104C	p.Q35P	22.9	0.01	.	.	.	
4	165103021	165103021	G	A	TMEM192	.	NM_001100389	c.C103T	p.Q35X	35	0.01	.	.	.	
22	19210459	19210459	C	T	CLTCL1	rs781878409	NM_007098	c.G3116A	p.R1039Q	32	0.01	.	.	1 × 10^−3^	
22	19219929	19219929	C	T	CLTCL1	rs188611399	NM_007098	c.G2875A	p.V959I	25.5	0.01	.	2 × 10^−4^	1 × 10^−3^	KIRP (2)
22	19224006	19224006	T	G	CLTCL1	rs782728804	NM_007098	c.A2177C	p.D726A	29.5	0.01	.	.	9 × 10^−6^	
22	19226346	19226346	T	C	CLTCL1	rs201280856	NM_007098	c.A1820G	p.H607R	25.5	0.01	.	3 × 10^−4^	4 × 10^−4^	
22	19233264	19233264	C	A	CLTCL1	rs782774942	NM_007098	c.G1423T	p.A475S	23.6	0.01	.	.	6 × 10^−5^	
22	19234672	19234672	A	G	CLTCL1	.	NM_007098	c.T1004C	p.V335A	25.3	0.01	.	.	.	
11	129872957	129872957	G	A	NFRKB	.	NM_006165	c.C2765T	p.P922L	31	0.01	.	.	.	
11	129874521	129874521	G	A	NFRKB	rs200192480	NM_006165	c.C2113T	p.P705S	23.9	0.01	.	.	4 × 10^−5^	fNTMC (1 family)
11	129884816	129884816	G	A	NFRKB	rs755726394	NM_006165	c.C746T	p.A249V	22.9	0.01	.	.	6 × 10^−5^	
22	41117808	41117808	C	T	EP300	.	NM_001429	c.C716T	p.P239L	21.3	0.01	.	.	.	
22	41137724	41137724	C	T	EP300	.	NM_001429	c.C1694T	p.T565I	24.9	0.01	.	.	.	
22	41149147	41149147	C	T	EP300	rs201480900	NM_001429	c.C2351T	p.P784L	23.2	0.01	.	8 × 10^−5^	2 × 10^−4^	SKCM (1)
16	70663765	70663765	G	A	MTSS2	rs749003640	NM_138383	c.C2156T	p.P719L	24.5	0.01	.	2 × 10^−4^	1 × 10^−3^	KIRP (1)
16	70664615	70664615	T	C	MTSS2	rs147433916	NM_138383	c.A1454G	p.D485G	23.9	0.01	2 × 10^−3^	2 × 10^−4^	2 × 10^−3^	SKCM (1)
16	70665044	70665044	C	T	MTSS2	rs549028223	NM_138383	c.G1181A	p.R394Q	26.1	0.01	.	5 × 10^−5^	2 × 10^−3^	
16	70679820	70679820	C	G	MTSS2	rs768341867	NM_138383	c.G348C	p.K116N	29.1	0.01	.	.	9 ×10^−6^	
3	47046509	47046509	C	T	SETD2	rs766193321	NM_001349370	c.G6944A	p.G2315E	33	0.01	.	3 × 10^−5^	9 × 10^−6^	
3	47046543	47046543	G	T	SETD2	.	NM_001349370	c.C6910A	p.P2304T	25.9	0.01	.	.	.	
3	47084114	47084114	A	G	SETD2	rs148097513	NM_001349370	c.T5534C	p.M1845T	25.3	0.01	9 × 10^−4^	1 × 10^−3^	2 × 10^−3^	KIRC (3)–KIRP (1)–SKCM (1)
3	47121407	47121407	T	C	SETD2	rs114719990	NM_001349370	c.A3097G	p.T1033A	23.6	0.01	2 × 10^−3^	2 × 10^−3^	2 × 10^−3^	SKCM (3)–KIRP (1)
3	47123308	47123308	C	G	SETD2	.	NM_001349370	c.G1196C	p.R399T	25.8	0.01	.	.	.	
9	104114033	104114033	C	T	SMC2	.	NM_001042550	c.C1484T	p.T495I	20.6	0.01	.	.	.	
9	104125007	104125007	G	A	SMC2	rs147960477	NM_001042550	c.G2353A	p.A785T	23	0.02	9 × 10^−4^	2 × 10^−4^	9 × 10^−4^	
9	104139220	104139220	A	G	SMC2	.	NM_001042550	c.A3499G	p.T1167A	23.9	0.01	.	.	.	
20	2706020	2706020	G	A	EBF4	rs202097996	NM_001110514	c.G329A	p.R110Q	21	0.01	9 × 10^−4^	6 × 10^−5^	1 × 10^−4^	
20	2706211	2706211	C	A	EBF4	.	NM_001110514	c.C349A	p.L117M	23.3	0.01	.	.	.	
20	2755749	2755749	G	A	EBF4	rs369331115	NM_001110514	c.G1651A	p.A551T	32	0.01	.	.	.	

Variants are ordered by ascending *q*-values of the candidate gene they belong to, that is, the Benjamini–Hochberg corrected ProxECAT-weighted statistics [47] from the gene-based test of enrichment in rare (AF ≤ 0.25%) exonic variants predicted to be deleterious in our series of 46 cases with CMM and RCC compared to external controls. Within a given gene, variants are ordered by ascending genome positions in GRCh38 (ANNOVAR annotations) [41]. * Occurrences in RCC and/or CMM TCGA series (KIRC, kidney renal clear cell carcinoma, N = 344; KIRP, kidney renal papillary cell carcinoma, N = 289; SKCM, skin cutaneous melanoma, N = 470—https://www.cancer.gov/tcga accessed on 25 January 2021) and/or familial cancer series (fNTMC, familial non-medullary thyroid cancer) [67], with the number of occurrence(s) between brackets for TCGA series and the number of affected families in which the variant segregates for familial cancer series. CADD: Combined Annotation Dependent Depletion score [45]; AF: allele frequency; AF_cases: AF in internal cases (N = 46); AF_FrEx: AF in the French reference panel ‘French Exome Project’ (N = 574, http://lysine.univ-brest.fr/FrExAC accessed on 25 January 2021); AF_controls: AF in the gnomAD non cancer samples [43,44] of north-western European ancestry (used as external controls in ProxECAT enrichment test, N = 19,751); AF_popmax: highest AF across all gnomAD v2.1.1 outbred populations exome (N = 125,748) and genome data (N = 15,708). An extended version of this table, including pseudonymized patient identifiers, is available as Supplementary Table S4.

**Table 5:** Biological pathways associated with the 13 candidate susceptibility genes identified in 46 French cases diagnosed with both CMM and RCC.

Pathway ID	Pathway Description	*q*-Value *	Number of Genes in Pathway	Candidate Genes in Pathway
KEGG:05215	Prostate cancer	2.8 × 10^−3^	97	*PIK3CD*, *MTOR*, *EP300*
KEGG:04066	HIF-1 signaling pathway	4 × 10^−3^	109	*PIK3CD*, *MTOR*, *EP300*
KEGG:04935	Growth hormone synthesis, secretion and action	5.1 × 10^−3^	118	*PIK3CD*, *MTOR*, *EP300*
KEGG:04919	Thyroid hormone signaling pathway	5.5 × 10^−3^	121	*PIK3CD*, *MTOR*, *EP300*
WP:WP4018	Pathways in clear cell renal cell carcinoma	7.7 × 10^−3^	86	*MTOR*, *EP300*, *SETD2*
KEGG:04630	JAK-STAT signaling pathway	1.3 × 10^−2^	162	*PIK3CD*, *MTOR*, *EP300*
KEGG:05164	Influenza A	1.5 × 10^−2^	169	*PIK3CD*, *RAE1*, *EP300*
WP:WP3287	Overview of nanoparticle effects	1.6 × 10^−2^	19	*PIK3CD*, *NFRKB*
KEGG:05167	Kaposi sarcoma-associated herpesvirus infection	2.2 × 10^−2^	193	*PIK3CD*, *MTOR*, *EP300*
WP:WP4217	Ebola Virus Pathway on Host	2.6 × 10^−2^	129	*PIK3CD*, *CLTCL1*, *EP300*
KEGG:04930	Type II diabetes mellitus	3.1 × 10^−2^	45	*PIK3CD*, *MTOR*
WP:WP4874	CAMKK2 Pathway	5 × 10^−2^	33	*MTOR*, *EP300*
WP:WP4241	Type 2 papillary renal cell carcinoma	5.3 × 10^−2^	34	*EP300*, *SETD2*
KEGG:04213	Longevity regulating pathway—multiple species	5.7 × 10^−2^	61	*PIK3CD*, *MTOR*
KEGG:05221	Acute myeloid leukemia	6.9 × 10^−2^	67	*PIK3CD*, *MTOR*
KEGG:05211	Renal cell carcinoma	7.1 × 10^−2^	68	*PIK3CD*, *EP300*
KEGG:05230	Central carbon metabolism in cancer	7.5 × 10^−2^	70	*PIK3CD*, *MTOR*
KEGG:05016	Huntington disease	8.4 × 10^−2^	306	*MTOR*, *CLTCL1*, *EP300*
KEGG:05214	Glioma	8.6 × 10^−2^	75	*PIK3CD*, *MTOR*
KEGG:05206	MicroRNAs in cancer	8.8 × 10^−2^	310	*PIK3CD*, *MTOR*, *EP300*
KEGG:05212	Pancreatic cancer	8.8 × 10^−2^	76	*PIK3CD*, *MTOR*
KEGG:05100	Bacterial invasion of epithelial cells	9.1 × 10^−2^	77	*PIK3CD*, *CLTCL1*
KEGG:01521	EGFR tyrosine kinase inhibitor resistance	9.5 × 10^−2^	79	*PIK3CD*, *MTOR*

* Top enriched pathways (*q* < 0.1) from Kyoto Encyclopedia of Genes and Genomes (KEGG) and WikiPathways (WP) using g:Profiler functional enrichment analyses with g:SCS multiple testing correction as per latest recommendations [53].

## Data Availability

The datasets used and/or analyzed during the current study are available from the corresponding author on reasonable request.

## Supplementary Materials

The following are available online at https://www.mdpi.com/article/10.3390/cancers13092243/s1, Table S1: Clinical and genetic characteristics of the 46 exome-sequenced cases affected by both malignant melanoma and RCC, Table S2: ProxECAT results for 908 genes hosting at least two rare deleterious variants in cases, Table S3: Burden of rare deleterious variants identified from TCGA melanoma and kidney cancer series in the 13 candidate CMM and/or RCC susceptibility genes, Table S4: Set of 41 rare deleterious variants observed in the 13 candidate CMM and/or RCC susceptibility genes with additional annotations, File S1: Relevance of candidate susceptibility genes to cancer development. File S2. Functional organization of the proteins encoded by our 13 candidate susceptibility genes.

## References

[1] Siegel R.L., Miller K.D., Jemal A. (2019). Cancer Statistics, 2019. CA Cancer J. Clin..

[2] Bray F., Ferlay J., Soerjomataram I., Siegel R.L., Torre L.A., Jemal A. (2018). Global Cancer Statistics 2018: GLOBOCAN Estimates of Incidence and Mortality Worldwide for 36 Cancers in 185 Countries. CA Cancer J. Clin..

[3] Chang A.E., Karnell L.H., Menck H.R. (1998). The National Cancer Data Base Report on Cutaneous and Noncutaneous Melanoma: A Summary of 84,836 Cases from the Past Decade. The American College of Surgeons Commission on Cancer and the American Cancer Society. Cancer.

[4] Maubec E., Chaudru V., Mohamdi H., Grange F., Patard J.-J., Dalle S., Crickx B., Paillerets B.B., Demenais F., Avril M.-F. (2010). Characteristics of the Coexistence of Melanoma and Renal Cell Carcinoma. Cancer.

[5] Abern M.R., Tsivian M., Coogan C.L., Kaufman H.L., Polascik T.J. (2013). Characteristics of Patients Diagnosed with Both Melanoma and Renal Cell Cancer. Cancer Causes Control.

[6] Flynn M., Pickering L., Larkin J., Turajlic S. (2018). Immune-Checkpoint Inhibitors in Melanoma and Kidney Cancer: From Sequencing to Rational Selection. Ther. Adv. Med. Oncol..

[7] Leonardi G.C., Falzone L., Salemi R., Zanghì A., Spandidos D.A., Mccubrey J.A., Candido S., Libra M. (2018). Cutaneous Melanoma: From Pathogenesis to Therapy (Review). Int. J. Oncol..

[8] Scelo G., Larose T.L. (2018). Epidemiology and Risk Factors for Kidney Cancer. J. Clin. Oncol..

[9] Clement E., Lazar I., Muller C., Nieto L. (2017). Obesity and Melanoma: Could Fat Be Fueling Malignancy?. Pigment Cell Melanoma Res..

[10] Dusingize J.C., Olsen C.M., An J., Pandeya N., Law M.H., Thompson B.S., Goldstein A.M., Iles M.M., Webb P.M., Neale R.E. (2020). Body Mass Index and Height and Risk of Cutaneous Melanoma: Mendelian Randomization Analyses. Int. J. Epidemiol..

[11] Scelo G., Purdue M.P., Brown K.M., Johansson M., Wang Z., Eckel-Passow J.E., Ye Y., Hofmann J.N., Choi J., Foll M. (2017). Genome-Wide Association Study Identifies Multiple Risk Loci for Renal Cell Carcinoma. Nat. Commun..

[12] Lu Y., Ek W.E., Whiteman D., Vaughan T.L., Spurdle A.B., Easton D.F., Pharoah P.D., Thompson D.J., Dunning A.M., Hayward N.K. (2014). Most Common “sporadic” Cancers Have a Significant Germline Genetic Component. Hum. Mol. Genet..

[13] Nasti T.H., Timares L. (2015). MC1R, Eumelanin and Pheomelanin: Their Role in Determining the Susceptibility to Skin Cancer. Photochem. Photobiol..

[14] Herraiz C., Garcia-Borron J.C., Jiménez-Cervantes C., Olivares C. (2017). MC1R Signaling. Intracellular Partners and Pathophysiological Implications. Biochim. Biophys. Acta Mol. Basis Dis..

[15] Robles-Espinoza C.D., Roberts N.D., Chen S., Leacy F.P., Alexandrov L.B., Pornputtapong N., Halaban R., Krauthammer M., Cui R., Timothy Bishop D. (2016). Germline MC1R Status Influences Somatic Mutation Burden in Melanoma. Nat. Commun..

[16] Williams P.F., Olsen C.M., Hayward N.K., Whiteman D.C. (2011). Melanocortin 1 Receptor and Risk of Cutaneous Melanoma: A Meta-Analysis and Estimates of Population Burden. Int. J. Cancer.

[17] Kiezun A., Garimella K., Do R., Stitziel N.O., Neale B.M., McLaren P.J., Gupta N., Sklar P., Sullivan P.F., Moran J.L. (2012). Exome Sequencing and the Genetic Basis of Complex Traits. Nat. Genet..

[18] Read J., Wadt K.A.W., Hayward N.K. (2016). Melanoma Genetics. J. Med. Genet..

[19] Goding C.R., Arnheiter H. (2019). MITF-the First 25 Years. Genes Dev..

[20] Phelep A., Laouari D., Bharti K., Burtin M., Tammaccaro S., Garbay S., Nguyen C., Vasseur F., Blanc T., Berissi S. (2017). MITF—A Controls Branching Morphogenesis and Nephron Endowment. PLoS Genet..

[21] Bertolotto C., Lesueur F., Giuliano S., Strub T., de Lichy M., Bille K., Dessen P., d’Hayer B., Mohamdi H., Remenieras A. (2011). A SUMOylation-Defective MITF Germline Mutation Predisposes to Melanoma and Renal Carcinoma. Nature.

[22] Walpole S., Pritchard A.L., Cebulla C.M., Pilarski R., Stautberg M., Davidorf F.H., de la Fouchardière A., Cabaret O., Golmard L., Stoppa-Lyonnet D. (2018). Comprehensive Study of the Clinical Phenotype of Germline BAP1 Variant-Carrying Families Worldwide. J. Natl. Cancer Inst..

[23] Louie B.H., Kurzrock R. (2020). BAP1: Not Just a BRCA1-Associated Protein. Cancer Treat. Rev..

[24] Bedogni B., Powell M.B. (2006). Skin Hypoxia: A Promoting Environmental Factor in Melanomagenesis. Cell Cycle.

[25] Zou A.-P., Cowley A.W. (2003). Reactive Oxygen Species and Molecular Regulation of Renal Oxygenation. Acta Physiol. Scand..

[26] Tempé D., Piechaczyk M., Bossis G. (2008). SUMO under Stress. Biochem. Soc. Trans..

[27] Bonet C., Luciani F., Ottavi J.-F., Leclerc J., Jouenne F.-M., Boncompagni M., Bille K., Hofman V., Bossis G., Marco de Donatis G. (2017). Deciphering the Role of Oncogenic MITFE318K in Senescence Delay and Melanoma Progression. J. Natl. Cancer Inst..

[28] Jafri M., Wake N.C., Ascher D.B., Pires D.E.V., Gentle D., Morris M.R., Rattenberry E., Simpson M.A., Trembath R.C., Weber A. (2015). Germline Mutations in the CDKN2B Tumor Suppressor Gene Predispose to Renal Cell Carcinoma. Cancer Discov..

[29] Hebert L., Bellanger D., Guillas C., Campagne A., Dingli F., Loew D., Fievet A., Jacquemin V., Popova T., Jean D. (2017). Modulating BAP1 Expression Affects ROS Homeostasis, Cell Motility and Mitochondrial Function. Oncotarget.

[30] Lesueur F., de Lichy M., Barrois M., Durand G., Bombled J., Avril M.-F., Chompret A., Boitier F., Lenoir G.M., French Familial Melanoma Study Group (2008). The Contribution of Large Genomic Deletions at the CDKN2A Locus to the Burden of Familial Melanoma. Br. J. Cancer.

[31] Li H., Durbin R. (2009). Fast and Accurate Short Read Alignment with Burrows-Wheeler Transform. Bioinformatics.

[32] DePristo M.A., Banks E., Poplin R., Garimella K.V., Maguire J.R., Hartl C., Philippakis A.A., del Angel G., Rivas M.A., Hanna M. (2011). A Framework for Variation Discovery and Genotyping Using Next-Generation DNA Sequencing Data. Nat. Genet..

[33] Cingolani P., Platts A., Wang L.L., Coon M., Nguyen T., Wang L., Land S.J., Lu X., Ruden D.M. (2012). A Program for Annotating and Predicting the Effects of Single Nucleotide Polymorphisms, SnpEff: SNPs in the Genome of Drosophila Melanogaster Strain W1118; Iso-2; Iso-3. Fly.

[34] Richards S., Aziz N., Bale S., Bick D., Das S., Gastier-Foster J., Grody W.W., Hegde M., Lyon E., Spector E. (2015). Standards and Guidelines for the Interpretation of Sequence Variants: A Joint Consensus Recommendation of the American College of Medical Genetics and Genomics and the Association for Molecular Pathology. Genet. Med..

[35] Wendt J., Mueller C., Rauscher S., Fae I., Fischer G., Okamoto I. (2018). Contributions by MC1R Variants to Melanoma Risk in Males and Females. JAMA Derm..

[36] Morgan M.D., Pairo-Castineira E., Rawlik K., Canela-Xandri O., Rees J., Sims D., Tenesa A., Jackson I.J. (2018). Genome-Wide Study of Hair Colour in UK Biobank Explains Most of the SNP Heritability. Nat. Commun..

[37] Di Tommaso P., Chatzou M., Floden E.W., Barja P.P., Palumbo E., Notredame C. (2017). Nextflow Enables Reproducible Computational Workflows. Nat. Biotechnol..

[38] Tarasov A., Vilella A.J., Cuppen E., Nijman I.J., Prins P. (2015). Sambamba: Fast Processing of NGS Alignment Formats. Bioinformatics.

[39] Van der Auwera G.A., Carneiro M.O., Hartl C., Poplin R., Del Angel G., Levy-Moonshine A., Jordan T., Shakir K., Roazen D., Thibault J. (2013). From FastQ Data to High Confidence Variant Calls: The Genome Analysis Toolkit Best Practices Pipeline. Curr. Protoc. Bioinform..

[40] Chang C.C., Chow C.C., Tellier L.C., Vattikuti S., Purcell S.M., Lee J.J. (2015). Second-Generation PLINK: Rising to the Challenge of Larger and Richer Datasets. Gigascience.

[41] Wang K., Li M., Hakonarson H. (2010). ANNOVAR: Functional Annotation of Genetic Variants from High-Throughput Sequencing Data. Nucleic Acids Res..

[42] Wigginton J.E., Cutler D.J., Abecasis G.R. (2005). A Note on Exact Tests of Hardy-Weinberg Equilibrium. Am. J. Hum. Genet..

[43] Lek M., Karczewski K.J., Minikel E.V., Samocha K.E., Banks E., Fennell T., O’Donnell-Luria A.H., Ware J.S., Hill A.J., Cummings B.B. (2016). Analysis of Protein-Coding Genetic Variation in 60,706 Humans. Nature.

[44] Karczewski K.J., Francioli L.C., Tiao G., Cummings B.B., Alföldi J., Wang Q., Collins R.L., Laricchia K.M., Ganna A., Birnbaum D.P. (2020). The Mutational Constraint Spectrum Quantified from Variation in 141,456 Humans. Nature.

[45] Rentzsch P., Witten D., Cooper G.M., Shendure J., Kircher M. (2019). CADD: Predicting the Deleteriousness of Variants throughout the Human Genome. Nucleic Acids Res..

[46] Landrum M.J., Lee J.M., Benson M., Brown G.R., Chao C., Chitipiralla S., Gu B., Hart J., Hoffman D., Jang W. (2018). ClinVar: Improving Access to Variant Interpretations and Supporting Evidence. Nucleic Acids Res..

[47] Hendricks A.E., Billups S.C., Pike H.N.C., Farooqi I.S., Zeggini E., Santorico S.A., Barroso I., Dupuis J. (2018). ProxECAT: Proxy External Controls Association Test. A New Case-Control Gene Region Association Test Using Allele Frequencies from Public Controls. PLoS Genet..

[48] Jiang Y., Epstein M.P., Conneely K.N. (2013). Assessing the Impact of Population Stratification on Association Studies of Rare Variation. Hum. Hered..

[49] Benjamini Y., Hochberg Y. (1995). Controlling the False Discovery Rate: A Practical and Powerful Approach to Multiple Testing. J. R. Stat. Soc. Ser. B (Methodol.).

[50] Wang Z., Wei Y., Zhang R., Su L., Gogarten S.M., Liu G., Brennan P., Field J.K., McKay J.D., Lissowska J. (2018). Multi-Omics Analysis Reveals a HIF Network and Hub Gene EPAS1 Associated with Lung Adenocarcinoma. EBioMedicine.

[51] Husson T., Duboc J.-B., Quenez O., Charbonnier C., Rotharmel M., Cuenca M., Jegouzo X., Richard A.-C., Frebourg T., Deleuze J.-F. (2018). Identification of Potential Genetic Risk Factors for Bipolar Disorder by Whole-Exome Sequencing. Transl. Psychiatry.

[52] Robinson J.T., Thorvaldsdóttir H., Wenger A.M., Zehir A., Mesirov J.P. (2017). Variant Review with the Integrative Genomics Viewer. Cancer Res..

[53] Raudvere U., Kolberg L., Kuzmin I., Arak T., Adler P., Peterson H., Vilo J. (2019). g: Profiler: A Web Server for Functional Enrichment Analysis and Conversions of Gene Lists (2019 Update). Nucleic Acids Res..

[54] Huang K.-L., Mashl R.J., Wu Y., Ritter D.I., Wang J., Oh C., Paczkowska M., Reynolds S., Wyczalkowski M.A., Oak N. (2018). Pathogenic Germline Variants in 10,389 Adult Cancers. Cell.

[55] Rimmer A., Phan H., Mathieson I., Iqbal Z., Twigg S.R.F., Wilkie A.O.M., McVean G., Lunter G., WGS500 Consortium (2014). Integrating Mapping-, Assembly- and Haplotype-Based Approaches for Calling Variants in Clinical Sequencing Applications. Nat. Genet..

[56] He H., Li W., Comiskey D.F., Liyanarachchi S., Nieminen T.T., Wang Y., DeLap K.E., Brock P., de la Chapelle A. (2020). A Truncating Germline Mutation of TINF2 in Individuals with Thyroid Cancer or Melanoma Results in Longer Telomeres. Thyroid.

[57] Maher E.R. (2018). Hereditary Renal Cell Carcinoma Syndromes: Diagnosis, Surveillance and Management. World J. Urol..

[58] Schmidt L.S., Linehan W.M. (2018). FLCN: The Causative Gene for Birt-Hogg-Dubé Syndrome. Gene.

[59] Aoude L.G., Pritchard A.L., Robles-Espinoza C.D., Wadt K., Harland M., Choi J., Gartside M., Quesada V., Johansson P., Palmer J.M. (2015). Nonsense Mutations in the Shelterin Complex Genes ACD and TERF2IP in Familial Melanoma. J. Natl. Cancer Inst..

[60] Pastorino L., Andreotti V., Dalmasso B., Vanni I., Ciccarese G., Mandalà M., Spadola G., Pizzichetta M.A., Ponti G., Tibiletti M.G. (2020). Insights into Genetic Susceptibility to Melanoma by Gene Panel Testing: Potential Pathogenic Variants in ACD, ATM, BAP1, and POT1. Cancers.

[61] Malińska K., Deptuła J., Rogoża-Janiszewska E., Górski B., Scott R., Rudnicka H., Kashyap A., Domagała P., Hybiak J., Masojć B. (2020). Constitutional Variants in POT1, TERF2IP, and ACD Genes in Patients with Melanoma in the Polish Population. Eur. J. Cancer Prev. Off. J. Eur. Cancer Prev. Organ..

[62] Machiela M.J., Hofmann J.N., Carreras-Torres R., Brown K.M., Johansson M., Wang Z., Foll M., Li P., Rothman N., Savage S.A. (2017). Genetic Variants Related to Longer Telomere Length Are Associated with Increased Risk of Renal Cell Carcinoma. Eur. Urol..

[63] Inoki K., Corradetti M.N., Guan K.-L. (2005). Dysregulation of the TSC-MTOR Pathway in Human Disease. Nat. Genet..

[64] Carlo M.I., Hakimi A.A., Stewart G.D., Bratslavsky G., Brugarolas J., Chen Y.-B., Linehan W.M., Maher E.R., Merino M.J., Offit K. (2019). Familial Kidney Cancer: Implications of New Syndromes and Molecular Insights. Eur. Urol..

[65] Rosengren T., Nanhoe S., de Almeida L.G.D., Schönewolf-Greulich B., Larsen L.J., Hey C.A.B., Dunø M., Ek J., Risom L., Nellist M. (2020). Mutational Analysis of TSC1 and TSC2 in Danish Patients with Tuberous Sclerosis Complex. Sci. Rep..

[66] Chen R., Zhao W.-Q., Fang C., Yang X., Ji M. (2020). Histone Methyltransferase SETD2: A Potential Tumor Suppressor in Solid Cancers. J. Cancer.

[67] Wang Y., Liyanarachchi S., Miller K.E., Nieminen T.T., Comiskey D.F., Li W., Brock P., Symer D.E., Akagi K., DeLap K.E. (2019). Identification of Rare Variants Predisposing to Thyroid Cancer. Thyroid.

[68] Riazalhosseini Y., Lathrop M. (2016). Precision Medicine from the Renal Cancer Genome. Nat. Rev. Nephrol..

[69] Martens M., Ammar A., Riutta A., Waagmeester A., Slenter D.N., Hanspers K., A Miller R., Digles D., Lopes E.N., Ehrhart F. (2020). WikiPathways: Connecting Communities. Nucleic Acids Res..

[70] Kanehisa M., Sato Y., Furumichi M., Morishima K., Tanabe M. (2019). New Approach for Understanding Genome Variations in KEGG. Nucleic Acids Res..

[71] Muthukrishnan S.D., Alvarado A.G., Kornblum H.I. (2018). Building Bonds: Cancer Stem Cells Depend on Their Progeny to Drive Tumor Progression. Cell Stem Cell.

[72] Parker H., Rose-Zerilli M.J.J., Larrayoz M., Clifford R., Edelmann J., Blakemore S., Gibson J., Wang J., Ljungström V., Wojdacz T.K. (2016). Genomic Disruption of the Histone Methyltransferase SETD2 in Chronic Lymphocytic Leukaemia. Leukemia.

[73] Byun J., Schwartz A.G., Lusk C., Wenzlaff A.S., de Andrade M., Mandal D., Gaba C., Yang P., You M., Kupert E.Y. (2018). Genome-Wide Association Study of Familial Lung Cancer. Carcinogenesis.

[74] Yokoyama S., Woods S.L., Boyle G.M., Aoude L.G., MacGregor S., Zismann V., Gartside M., Cust A.E., Haq R., Harland M. (2011). A Novel Recurrent Mutation in MITF Predisposes to Familial and Sporadic Melanoma. Nature.

[75] Potrony M., Puig-Butille J.A., Aguilera P., Badenas C., Tell-Marti G., Carrera C., Javier Del Pozo L., Conejo-Mir J., Malvehy J., Puig S. (2016). Prevalence of MITF p.E318K in Patients With Melanoma Independent of the Presence of CDKN2A Causative Mutations. JAMA Dermatol..

[76] Potjer T.P., Bollen S., Grimbergen A.J.E.M., van Doorn R., Gruis N.A., van Asperen C.J., Hes F.J., van der Stoep N. (2019). Dutch Working Group for Clinical Oncogenetics Multigene Panel Sequencing of Established and Candidate Melanoma Susceptibility Genes in a Large Cohort of Dutch Non-CDKN2A/CDK4 Melanoma Families. Int. J. Cancer.

[77] Gromowski T., Masojć B., Scott R.J., Cybulski C., Górski B., Kluźniak W., Paszkowska-Szczur K., Rozmiarek A., Dębniak B., Maleszka R. (2014). Prevalence of the E318K and V320I MITF Germline Mutations in Polish Cancer Patients and Multiorgan Cancer Risk-a Population-Based Study. Cancer Genet..

[78] Stoehr C.G., Walter B., Denzinger S., Ghiorzo P., Sturm R.A., Hinze R., Moch H., Junker K., Hartmann A., Stoehr R. (2016). The Microphthalmia-Associated Transcription Factor p.E318K Mutation Does Not Play a Major Role in Sporadic Renal Cell Tumors from Caucasian Patients. Pathobiology.

[79] Smith P.S., West H., Whitworth J., Castle B., Sansbury F.H., Warren A.Y., Woodward E.R., Tischkowitz M., Maher E.R. (2021). Pathogenic Germline Variants in Patients with Features of Hereditary Renal Cell Carcinoma: Evidence for Further Locus Heterogeneity. Genes Chromosomes Cancer.

[80] Lang M., Vocke C.D., Ricketts C.J., Metwalli A.R., Ball M.W., Schmidt L.S., Linehan W.M. (2020). Clinical and Molecular Characterization of Microphthalmia-Associated Transcription Factor (MITF)-Related Renal Cell Carcinoma. Urology.

[81] Chau C., van Doorn R., van Poppelen N.M., van der Stoep N., Mensenkamp A.R., Sijmons R.H., van Paassen B.W., van den Ouweland A.M.W., Naus N.C., van der Hout A.H. (2019). Families with BAP1-Tumor Predisposition Syndrome in The Netherlands: Path to Identification and a Proposal for Genetic Screening Guidelines. Cancers.

[82] Cocciolone R.A., Crotty K.A., Andrews L., Haass N.K., Moloney F.J. (2010). Multiple Desmoplastic Melanomas in Birt-Hogg-Dubé Syndrome and a Proposed Signaling Link between Folliculin, the MTOR Pathway, and Melanoma Susceptibility. Arch. Dermatol..

[83] Sattler E.C., Ertl-Wagner B., Pellegrini C., Peris K., Reithmair M., Schädle N., Ruzicka T., Steinlein O.K. (2018). Cutaneous Melanoma in Birt-Hogg-Dubé Syndrome: Part of the Clinical Spectrum?. Br. J. Dermatol..

[84] Ricketts C.J., De Cubas A.A., Fan H., Smith C.C., Lang M., Reznik E., Bowlby R., Gibb E.A., Akbani R., Beroukhim R. (2018). The Cancer Genome Atlas Comprehensive Molecular Characterization of Renal Cell Carcinoma. Cell Rep..

[85] Tan M.-H., Mester J.L., Ngeow J., Rybicki L.A., Orloff M.S., Eng C. (2012). Lifetime Cancer Risks in Individuals with Germline PTEN Mutations. Clin. Cancer Res..

[86] Jiang N., Dai Q., Su X., Fu J., Feng X., Peng J. (2020). Role of PI3K/AKT Pathway in Cancer: The Framework of Malignant Behavior. Mol. Biol. Rep..

[87] Pópulo H., Lopes J.M., Soares P. (2012). The MTOR Signalling Pathway in Human Cancer. Int. J. Mol. Sci..

[88] Stahl J.M., Cheung M., Sharma A., Trivedi N.R., Shanmugam S., Robertson G.P. (2003). Loss of PTEN Promotes Tumor Development in Malignant Melanoma. Cancer Res..

[89] Keppler-Noreuil K.M., Parker V.E.R., Darling T.N., Martinez-Agosto J.A. (2016). Somatic Overgrowth Disorders of the PI3K/AKT/MTOR Pathway & Therapeutic Strategies. Am. J. Med. Genet. C Semin. Med. Genet..

[90] Yehia L., Ngeow J., Eng C. (2019). PTEN-Opathies: From Biological Insights to Evidence-Based Precision Medicine. J. Clin. Investig..

[91] Zöllner J.P., Franz D.N., Hertzberg C., Nabbout R., Rosenow F., Sauter M., Schubert-Bast S., Wiemer-Kruel A., Strzelczyk A. (2020). A Systematic Review on the Burden of Illness in Individuals with Tuberous Sclerosis Complex (TSC). Orphanet J. Rare Dis..

[92] Guo H., German P., Bai S., Barnes S., Guo W., Qi X., Lou H., Liang J., Jonasch E., Mills G.B. (2015). The PI3K/AKT Pathway and Renal Cell Carcinoma. J. Genet. Genom. Yi Chuan Xue Bao.

[93] Chamcheu J.C., Roy T., Uddin M.B., Banang-Mbeumi S., Chamcheu R.-C.N., Walker A.L., Liu Y.-Y., Huang S. (2019). Role and Therapeutic Targeting of the PI3K/Akt/MTOR Signaling Pathway in Skin Cancer: A Review of Current Status and Future Trends on Natural and Synthetic Agents Therapy. Cells.

[94] Chappell J.C., Payne L.B., Rathmell W.K. (2019). Hypoxia, Angiogenesis, and Metabolism in the Hereditary Kidney Cancers. J. Clin. Investig..

[95] Kim E., Zucconi B.E., Wu M., Nocco S.E., Meyers D.J., McGee J.S., Venkatesh S., Cohen D.L., Gonzalez E.C., Ryu B. (2019). MITF Expression Predicts Therapeutic Vulnerability to P300 Inhibition in Human Melanoma. Cancer Res..

[96] Milani D., Manzoni F.M.P., Pezzani L., Ajmone P., Gervasini C., Menni F., Esposito S. (2015). Rubinstein-Taybi Syndrome: Clinical Features, Genetic Basis, Diagnosis, and Management. Ital. J. Pediatr..

[97] Arany Z., Huang L.E., Eckner R., Bhattacharya S., Jiang C., Goldberg M.A., Bunn H.F., Livingston D.M. (1996). An Essential Role for P300/CBP in the Cellular Response to Hypoxia. Proc. Natl. Acad. Sci. USA.

[98] Yang G., Shi R., Zhang Q. (2020). Hypoxia and Oxygen-Sensing Signaling in Gene Regulation and Cancer Progression. Int. J. Mol. Sci..

[99] Martínez-García M.Á., Riveiro-Falkenbach E., Rodríguez-Peralto J.L., Nagore E., Martorell-Calatayud A., Campos-Rodríguez F., Farré R., Hernández Blasco L., Bañuls Roca J., Chiner Vives E. (2017). A Prospective Multicenter Cohort Study of Cutaneous Melanoma: Clinical Staging and Potential Associations with HIF-1α and VEGF Expressions. Melanoma Res..

[100] Christensen M.B., Wadt K., Jensen U.B., Lautrup C.K., Bojesen A., Krogh L.N., van Overeem Hansen T., Gerdes A.-M. (2019). Exploring the Hereditary Background of Renal Cancer in Denmark. PLoS ONE.

[101] Rotunno M., Barajas R., Clyne M., Hoover E., Simonds N.I., Lam T.K., Mechanic L.E., Goldstein A.M., Gillanders E.M. (2020). A Systematic Literature Review of Whole Exome and Genome Sequencing Population Studies of Genetic Susceptibility to Cancer. Cancer Epidemiol. Biomark. Prev..

[102] Artomov M., Stratigos A.J., Kim I., Kumar R., Lauss M., Reddy B.Y., Miao B., Daniela Robles-Espinoza C., Sankar A., Njauw C.-N. (2017). Rare Variant, Gene-Based Association Study of Hereditary Melanoma Using Whole-Exome Sequencing. J. Natl. Cancer Inst..

[103] Galvan A., Ioannidis J.P.A., Dragani T.A. (2010). Beyond Genome-Wide Association Studies: Genetic Heterogeneity and Individual Predisposition to Cancer. Trends Genet. TIG.

[104] Nielsen F.C., van Overeem Hansen T., Sørensen C.S. (2016). Hereditary Breast and Ovarian Cancer: New Genes in Confined Pathways. Nat. Rev. Cancer.

[105] Babu J.R., Jeganathan K.B., Baker D.J., Wu X., Kang-Decker N., van Deursen J.M. (2003). Rae1 Is an Essential Mitotic Checkpoint Regulator That Cooperates with Bub3 to Prevent Chromosome Missegregation. J. Cell Biol..

[106] Walker C., Burggren W. (2020). Remodeling the Epigenome and (Epi)Cytoskeleton: A New Paradigm for Co-Regulation by Methylation. J. Exp. Biol..

[107] Royle S.J. (2012). The Role of Clathrin in Mitotic Spindle Organisation. J. Cell Sci..

[108] Gorodetska I., Kozeretska I., Dubrovska A. (2019). BRCA Genes: The Role in Genome Stability, Cancer Stemness and Therapy Resistance. J. Cancer.

[109] Chan S.H., Ngeow J. (2017). Germline Mutation Contribution to Chromosomal Instability. Endocr. Relat. Cancer.

[110] Gerstenblith M.R., Goldstein A.M., Fargnoli M.C., Peris K., Landi M.T. (2007). Comprehensive Evaluation of Allele Frequency Differences of MC1R Variants across Populations. Hum. Mutat..

[111] Cao J., Wan L., Hacker E., Dai X., Lenna S., Jimenez-Cervantes C., Wang Y., Leslie N.R., Xu G.X., Widlund H.R. (2013). MC1R Is a Potent Regulator of PTEN after UV Exposure in Melanocytes. Mol. Cell.

[112] Gong R. (2011). The Renaissance of Corticotropin Therapy in Proteinuric Nephropathies. Nat. Rev. Nephrol..

[113] Pośpiech E., Ligęza J., Wilk W., Gołas A., Jaszczyński J., Stelmach A., Ryś J., Blecharczyk A., Wojas-Pelc A., Jura J. (2015). Variants of SCARB1 and VDR Involved in Complex Genetic Interactions May Be Implicated in the Genetic Susceptibility to Clear Cell Renal Cell Carcinoma. Biomed. Res. Int..

[114] Zhang B., Sun N., Mu X., Zhi L., Zhai L., Jiang Y., Fu Z., Yao Z. (2017). G Protein Alpha S Subunit Promotes Cell Proliferation of Renal Cell Carcinoma with Involvement of Protein Kinase A Signaling. DNA Cell Biol..

[115] Carlson J., Locke A.E., Flickinger M., Zawistowski M., Levy S., Myers R.M., Boehnke M., Kang H.M., Scott L.J., Li J.Z. (2018). Extremely Rare Variants Reveal Patterns of Germline Mutation Rate Heterogeneity in Humans. Nat. Commun..

