# Supplementary material for: The PI3K/mTOR Pathway Is Targeted by Rare Germline Variants in Patients with Both Melanoma and Renal Cell Carcinoma
Source: Cancers (Basel). 2021 May 7;13(9):2243. doi: 10.3390/cancers13092243 (PMC8125037; doi:10.3390/cancers13092243)
Supplement: Supplementary file 1 [file cancers-13-02243-s001.zip › Hubert_Suybeng_MelanomaRCC_Supp_File1_proofread.docx]

**File S1.** Relevance of candidate susceptibility genes to cancer development.

- *PIK3CD*

*PIK3CD* is a phosphoinositide 3‐kinase gene encoding the p110δ subunit, one of the three existing catalytic subunits of class I PI3 kinases. PI3 kinases are essential components for the transduction of signal from tyrosine kinase receptors (RTKs), working antagonistically to the *PTEN* phosphatase, well described in human oncogenesis [1]. Changes in the catalytic activity of the PI3K complex are in particular associated with bidirectional regulations between the PI3K/Akt and MAP kinase pathways, which is highly relevant in somatic melanoma [2]. The other catalytic subunits acting downstream of RTKs are encoded by *PIK3CA* and *PIK3CB*, the first hosting an established genetic variation associated with both melanoma and RCC [3]. *PIK3CD* shows sequence and/or expression alterations in a growing list of cancers, including colorectal cancer [4], different types of lymphomas [5] [6] [7], glioblastoma [8], cervical cancer [9], and is today seen as a therapeutic target of great interest [3] [10]. Recent studies suggest that *PIK3CD* supports cell migration and invasion in colorectal and kidney cancer, in which it correlates with poor survival [4] [11]. The central role of the PI3K/AKT network in immune functions is emerging [12]. Germline mutations in *PIK3CD* are causally implicated in a variety of immunodeficiencies [13] [14] [15] [16], whereby an increased risk of malignant lymphoproliferation, and to a lesser extent non-hematopoietic malignancy, have been described [17] [18]. Of note, the *PIK3CD* germline variants identified in the present study are located in the terminal part of the ABD domain (File S2), which hosts rare germline variants causing Activated PI3K-δ Syndrome [19].

- *MTOR*

In the PI3K pathway, *MTOR* encodes a key kinase which integrates signals in response to various stimuli such as growth factor signaling, cellular nutrients (*i.e.*, amino acids, glucose and oxygen) and energy supply [20], and regulates many tumorigenesis-related transcription factors [21] [22]. *MTOR* has been found dysregulated or altered in both sporadic and familial cancers [21] [23] [24], and is an appealing candidate for therapeutic targeting [21] [25], including in RCC [26] and melanoma [27]. In RCC, mTOR dysregulation has been showed to induce neoplastic transformation through increased cell proliferation and survival. Its activation is associated with aggressive pathologic features and unfavorable outcome [28]. Also, *MTOR* nonsynonymous mutations are frequent in melanoma patients and may predict a worse prognosis [29]. Interestingly, the five variants affecting *PIK3CD* and *MTOR* are all located in three protein interaction regions (File S2). Possible interactions involve other PIKK family members associated with cancer, such as *ATM* and *PIK3R1*, and the MRE11-RAD50-NBN (MRN) complex, which is a master regulator of DNA damage response [30].

- *RAE1*

*RAE1* is involved in several nucleus-related processes, including nucleocytoplasmic transport and mitosis regulation. Haplo-insufficiency of *RAE1* impairs mitotic checkpoint function, promoting chromosomal instability and tumorigenesis [31]. Overexpression of *RAE1* has been shown to cause aggressive breast cancer phenotypes [32]. In addition, germline variants in its homologue *BUB3* may predispose to early-onset colorectal cancer [33]. *RAE1* also acts as a key mediator in a recently identified control mechanism of the MAPK pathway [34], which is deregulated in many cancers and is of particular importance in melanoma [35]. In RCC patients, the expression of *RAE1* in peripheral blood mononuclear cells is associated with better prognosis [36].

- *ZBTB21*

*ZBTB21* is a member of the Zinc finger and BTB/POZ domain containing transcription factor family. Its biological role is not yet elucidated. Other genes in the family have important roles in various cancers and regulate the transcription of target genes at play in melanoma and/or RCC [37] [38] [39].

- *ESAM*

*ESAM* encodes a cell adhesion molecule from the immunoglobulin superfamily localized at endothelial cell-cell contacts, suggestive of a possible role in angiogenesis. Its involvement in melanoma growth, migration and metastasis of has been experimentally demonstrated [40] [41].

- *TMEM192*

*TMEM192* encodes a transmembrane lysosomal protein whose function is not fully characterized. Some evidence suggests a role for *TMEM192* in the control of tumor growth, apoptosis and autophagy [42] [43]. The interaction between *TMEM192* and *RARRES1*, which exhibits tumor suppressive features in many cancers [44] [45] including melanoma [46], has been shown to promote autophagy in human cells [47].

- *CLTCL1*

*CLTCL1* encodes a clathrin heavy chain (CHC) isoform, known to play a key role in glucose uptake through the regulation of intracellular trafficking of the insulin-dependent glucose transporter GLUT4 [48]. More generally, clathrins contribute to mitotic spindle stabilization [49] [50] and to membrane trafficking of growth factor receptors [51], both processes mediating critical cancer pathways. Davila *et al.* [52] suggested that a dysregulation of the mitotic function of *CLTCL1* may cause somatic patterns of rearrangements in early tumor [52]. Initially uncovered as a tumor-suppressor candidate in meningioma cells [53], *CLTCL1* was later proposed as candidate susceptibility gene in familial lung squamous cancer [54]. Of note, recent evidence suggests a non-canonical role for *CLTC*, a close homologue of *CLTCL1*, in promoting cell proliferation and survival through EGFR/Akt/MAPK1 activation in liver cancer [55]. A link between *CLTC* and p53-mediated gene regulation in cancer, through the recruitment of p300 (encoded by *EP300*), has also been proposed [56] [57]. In addition, *CLTC* has been reported to be fused to *TFE3* (which belongs to the same family as *MITF*) in a pediatric Xp11.2 translocation RCC case with positivity for melanocytic markers [58].

- *NFRKB*

*NFRKB*, also known as *INO80G*, encodes a subunit of the INO80 chromatin-remodeling complex, which is involved in various chromosome-related functions essential for genome stability [59] and has documented roles in both melanoma [60] and RCC [61]. The INO80 complex is strongly connected with the mTOR pathway and regulates the expression of key signaling effectors of the pathway [62]. Evidence suggests that *BAP1*, which hosts germline variation associated with an increased risk of both cancers [63] [64], exerts a tumor suppressive action by stabilizing INO80 [65]. In addition, *NFRKB* has a rare functional domain allowing interaction with *BAP1*-like deubiquitinases [66] [67].

- *EP300*

*EP300*, together with its homologue and partner *CREBBP*, is a histone acetyltransferase gene with multiple cellular functions critical in human cancers [68] [69] [70] [71] [72] [73] [74], notably mediating the HIF pathway [75]. The involvement of *EP300* has been extensively documented in both melanoma [69] [74] [76] and RCC [77], including in minor [77] and extremely rare forms of RCC [78]. The exact functional contributions of *EP300* to cancers are various, complex and cell context dependent but evidence for both transcriptional and epigenetic regulations has been suggested in melanoma [79] and provided in RCC [80] [81].

- *MTSS2*

*MTSS2*, also known as Metastasis Suppressor 1-Like because of its homology with a gene identified as potential metastasis suppressor [82], has so far been associated with nervous system functions and disorders [83] [84]. The better characterized homologue MTSS1 has in particular been identified as a metastasis driver in melanocytes [85], as part of the Hedgehog pathway [86], which predispose to basal cell carcinomas of the skin [87], and as lymphoma-predisposing in the mouse [88]. A recent unpublished proteomics analysis by Kamireddy *et al.* (2020) from University California, San Diego, (<https://escholarship.org/uc/item/4d32m3d9>) identified *MTSS2* as a putative downstream substrate in the LKB1/AMPK pathway, which is connected with the mTOR pathway [89].

- *SETD2*

*SETD2* is located at 3p21.31, a hot spot region of deletion in RCC, and encodes a histone methyltransferase. *SETD2* is frequently mutated both in RCC [90] and in melanoma [91]. It has an established tumor-suppressive role through multiple cellular pathways such as transcription regulation, translation elongation and RNA splicing. *SETD2* depletion diminishes the expression of genes involved in metabolic pathways, with a phenotypic end point of decreased cell proliferation [92].

- *SMC2*

*SMC2* encodes a core subunit of the condensing complex, which plays essential roles in chromosome compaction and organization [93]. DNA damage-induced inhibition of *SMC2* [94] is mediated via the cell cycle checkpoint regulator *CHEK2*, itself involved in the susceptibility of many cancers [95]. Somatic alterations of *SMC2* have been described in several cancers [96] [97] [98], including melanoma [97]. In addition, *SMC2* has recently been proposed as candidate for pancreatic cancer susceptibility [99].

- *EBF4*

*EBF4* belongs to a family of four highly conserved DNA-binding transcription factors with tumor-suppressive roles in several cancers [100]. Its homologues *EBF1* and *EBF3* are able to interact with *EP300* [100]. In melanoma, *EBF3* has been proposed as an epigenetic driver of metastasis [101] and as a candidate melanoma susceptibility gene [102]. *EBF4* functions remain unknown, although it was recently associated with signaling pathways involved in breast cancer metastasis [103].

**Supplemental references**

1. Chalhoub, N.; Baker, S.J. PTEN and the PI3-Kinase Pathway in Cancer. *Annu Rev Pathol* **2009**, *4*, 127–150, doi:10.1146/annurev.pathol.4.110807.092311.

2. Russo, A.E.; Torrisi, E.; Bevelacqua, Y.; Perrotta, R.; Libra, M.; McCubrey, J.A.; Spandidos, D.A.; Stivala, F.; Malaponte, G. Melanoma: Molecular Pathogenesis and Emerging Target Therapies (Review). *Int J Oncol* **2009**, *34*, 1481–1489, doi:10.3892/ijo_00000277.

3. Arafeh, R.; Samuels, Y. PIK3CA in Cancer: The Past 30 Years. *Semin Cancer Biol* **2019**, *59*, 36–49, doi:10.1016/j.semcancer.2019.02.002.

4. Chen, J.-S.; Huang, J.-Q.; Luo, B.; Dong, S.-H.; Wang, R.-C.; Jiang, Z.-K.; Xie, Y.-K.; Yi, W.; Wen, G.-M.; Zhong, J.-F. PIK3CD Induces Cell Growth and Invasion by Activating AKT/GSK-3β/β-Catenin Signaling in Colorectal Cancer. *Cancer Sci* **2019**, *110*, 997–1011, doi:10.1111/cas.13931.

5. Dong, T.; Liu, Z.; Zhao, S.; Hu, C.; Liu, Y.; Ma, W.; Zhang, Q. The Expression of CD9 and PIK3CD Is Associated with Prognosis of Follicular Lymphoma. *J Cancer* **2015**, *6*, 1222–1229, doi:10.7150/jca.11279.

6. McKinney, M.; Moffitt, A.B.; Gaulard, P.; Travert, M.; De Leval, L.; Nicolae, A.; Raffeld, M.; Jaffe, E.S.; Pittaluga, S.; Xi, L.; et al. The Genetic Basis of Hepatosplenic T-Cell Lymphoma. *Cancer Discov* **2017**, *7*, 369–379, doi:10.1158/2159-8290.CD-16-0330.

7. Cui, W.; Zheng, S.; Li, X.; Ma, Y.; Sang, W.; Liu, M.; Zhang, W.; Zhou, X. PIK3CD Promoted Proliferation in Diffuse Large B Cell Lymphoma through Upregulation of C-Myc. *Tumour Biol* **2016**, *37*, 12767–12777, doi:10.1007/s13277-016-5225-5.

8. Pridham, K.J.; Varghese, R.T.; Sheng, Z. The Role of Class IA Phosphatidylinositol-4,5-Bisphosphate 3-Kinase Catalytic Subunits in Glioblastoma. *Front Oncol* **2017**, *7*, 312, doi:10.3389/fonc.2017.00312.

9. Cui, F.; Li, X.; Zhu, X.; Huang, L.; Huang, Y.; Mao, C.; Yan, Q.; Zhu, J.; Zhao, W.; Shi, H. MiR-125b Inhibits Tumor Growth and Promotes Apoptosis of Cervical Cancer Cells by Targeting Phosphoinositide 3-Kinase Catalytic Subunit Delta. *Cell Physiol Biochem* **2012**, *30*, 1310–1318, doi:10.1159/000343320.

10. Xenou, L.; Papakonstanti, E.A. P110δ PI3K as a Therapeutic Target of Solid Tumours. *Clin Sci (Lond)* **2020**, *134*, 1377–1397, doi:10.1042/CS20190772.

11. Fan, D.; Liu, Q.; Wu, F.; Liu, N.; Qu, H.; Yuan, Y.; Li, Y.; Gao, H.; Ge, J.; Xu, Y.; et al. Prognostic Significance of PI3K/AKT/ MTOR Signaling Pathway Members in Clear Cell Renal Cell Carcinoma. *PeerJ* **2020**, *8*, e9261, doi:10.7717/peerj.9261.

12. Fruman, D.A.; Chiu, H.; Hopkins, B.D.; Bagrodia, S.; Cantley, L.C.; Abraham, R.T. The PI3K Pathway in Human Disease. *Cell* **2017**, *170*, 605–635, doi:10.1016/j.cell.2017.07.029.

13. Sharfe, N.; Karanxha, A.; Dadi, H.; Merico, D.; Chitayat, D.; Herbrick, J.-A.; Freeman, S.; Grinstein, S.; Roifman, C.M. Dual Loss of P110δ PI3-Kinase and SKAP (KNSTRN) Expression Leads to Combined Immunodeficiency and Multisystem Syndromic Features. *J Allergy Clin Immunol* **2018**, *142*, 618–629, doi:10.1016/j.jaci.2017.10.033.

14. Jou, S.-T.; Chien, Y.-H.; Yang, Y.-H.; Wang, T.-C.; Shyur, S.-D.; Chou, C.-C.; Chang, M.-L.; Lin, D.-T.; Lin, K.-H.; Chiang, B.-L. Identification of Variations in the Human Phosphoinositide 3-Kinase P110delta Gene in Children with Primary B-Cell Immunodeficiency of Unknown Aetiology. *Int J Immunogenet* **2006**, *33*, 361–369, doi:10.1111/j.1744-313X.2006.00627.x.

15. Lucas, C.L.; Kuehn, H.S.; Zhao, F.; Niemela, J.E.; Deenick, E.K.; Palendira, U.; Avery, D.T.; Moens, L.; Cannons, J.L.; Biancalana, M.; et al. Dominant-Activating Germline Mutations in the Gene Encoding the PI(3)K Catalytic Subunit P110δ Result in T Cell Senescence and Human Immunodeficiency. *Nat Immunol* **2014**, *15*, 88–97, doi:10.1038/ni.2771.

16. Angulo, I.; Vadas, O.; Garçon, F.; Banham-Hall, E.; Plagnol, V.; Leahy, T.R.; Baxendale, H.; Coulter, T.; Curtis, J.; Wu, C.; et al. Phosphoinositide 3-Kinase δ Gene Mutation Predisposes to Respiratory Infection and Airway Damage. *Science* **2013**, *342*, 866–871, doi:10.1126/science.1243292.

17. Crank, M.C.; Grossman, J.K.; Moir, S.; Pittaluga, S.; Buckner, C.M.; Kardava, L.; Agharahimi, A.; Meuwissen, H.; Stoddard, J.; Niemela, J.; et al. Mutations in PIK3CD Can Cause Hyper IgM Syndrome (HIGM) Associated with Increased Cancer Susceptibility. *J Clin Immunol* **2014**, *34*, 272–276, doi:10.1007/s10875-014-0012-9.

18. Leven, E.A.; Maffucci, P.; Ochs, H.D.; Scholl, P.R.; Buckley, R.H.; Fuleihan, R.L.; Geha, R.S.; Cunningham, C.K.; Bonilla, F.A.; Conley, M.E.; et al. Hyper IgM Syndrome: A Report from the USIDNET Registry. *J Clin Immunol* **2016**, *36*, 490–501, doi:10.1007/s10875-016-0291-4.

19. Heurtier, L.; Lamrini, H.; Chentout, L.; Deau, M.-C.; Bouafia, A.; Rosain, J.; Plaza, J.-M.; Parisot, M.; Dumont, B.; Turpin, D.; et al. Mutations in the Adaptor-Binding Domain and Associated Linker Region of P110δ Cause Activated PI3K-δ Syndrome 1 (APDS1). *Haematologica* **2017**, *102*, e278–e281, doi:10.3324/haematol.2017.167601.

20. Dibble, C.C.; Manning, B.D. Signal Integration by MTORC1 Coordinates Nutrient Input with Biosynthetic Output. *Nat Cell Biol* **2013**, *15*, 555–564, doi:10.1038/ncb2763.

21. Chiarini, F.; Evangelisti, C.; McCubrey, J.A.; Martelli, A.M. Current Treatment Strategies for Inhibiting MTOR in Cancer. *Trends Pharmacol Sci* **2015**, *36*, 124–135, doi:10.1016/j.tips.2014.11.004.

22. Xu, K.; Liu, P.; Wei, W. MTOR Signaling in Tumorigenesis. *Biochim Biophys Acta* **2014**, *1846*, 638–654, doi:10.1016/j.bbcan.2014.10.007.

23. Tan, F.H.; Bai, Y.; Saintigny, P.; Darido, C. MTOR Signalling in Head and Neck Cancer: Heads Up. *Cells* **2019**, *8*, doi:10.3390/cells8040333.

24. He, J.; Wang, M.-Y.; Qiu, L.-X.; Zhu, M.-L.; Shi, T.-Y.; Zhou, X.-Y.; Sun, M.-H.; Yang, Y.-J.; Wang, J.-C.; Jin, L.; et al. Genetic Variations of MTORC1 Genes and Risk of Gastric Cancer in an Eastern Chinese Population. *Mol Carcinog* **2013**, *52 Suppl 1*, E70-79, doi:10.1002/mc.22013.

25. Hua, H.; Kong, Q.; Zhang, H.; Wang, J.; Luo, T.; Jiang, Y. Targeting MTOR for Cancer Therapy. *J Hematol Oncol* **2019**, *12*, 71, doi:10.1186/s13045-019-0754-1.

26. Wysocki, P.J. MTOR in Renal Cell Cancer: Modulator of Tumor Biology and Therapeutic Target. *Expert Rev Mol Diagn* **2009**, *9*, 231–241, doi:10.1586/erm.09.8.

27. Chamcheu, J.C.; Roy, T.; Uddin, M.B.; Banang-Mbeumi, S.; Chamcheu, R.-C.N.; Walker, A.L.; Liu, Y.-Y.; Huang, S. Role and Therapeutic Targeting of the PI3K/Akt/MTOR Signaling Pathway in Skin Cancer: A Review of Current Status and Future Trends on Natural and Synthetic Agents Therapy. *Cells* **2019**, *8*, doi:10.3390/cells8080803.

28. Rausch, S.; Schollenberger, D.; Hennenlotter, J.; Stühler, V.; Kruck, S.; Stenzl, A.; Bedke, J. MTOR and MTOR Phosphorylation Status in Primary and Metastatic Renal Cell Carcinoma Tissue: Differential Expression and Clinical Relevance. *J Cancer Res Clin Oncol* **2019**, *145*, 153–163, doi:10.1007/s00432-018-2775-5.

29. Kong, Y.; Si, L.; Li, Y.; Wu, X.; Xu, X.; Dai, J.; Tang, H.; Ma, M.; Chi, Z.; Sheng, X.; et al. Analysis of MTOR Gene Aberrations in Melanoma Patients and Evaluation of Their Sensitivity to PI3K-AKT-MTOR Pathway Inhibitors. *Clin Cancer Res* **2016**, *22*, 1018–1027, doi:10.1158/1078-0432.CCR-15-1110.

30. Bian, L.; Meng, Y.; Zhang, M.; Li, D. MRE11-RAD50-NBS1 Complex Alterations and DNA Damage Response: Implications for Cancer Treatment. *Mol Cancer* **2019**, *18*, 169, doi:10.1186/s12943-019-1100-5.

31. Babu, J.R.; Jeganathan, K.B.; Baker, D.J.; Wu, X.; Kang-Decker, N.; van Deursen, J.M. Rae1 Is an Essential Mitotic Checkpoint Regulator That Cooperates with Bub3 to Prevent Chromosome Missegregation. *J Cell Biol* **2003**, *160*, 341–353, doi:10.1083/jcb.200211048.

32. Oh, J.H.; Lee, J.-Y.; Yu, S.; Cho, Y.; Hur, S.; Nam, K.T.; Kim, M.H. RAE1 Mediated ZEB1 Expression Promotes Epithelial-Mesenchymal Transition in Breast Cancer. *Sci Rep* **2019**, *9*, 2977, doi:10.1038/s41598-019-39574-8.

33. Mur, P.; De Voer, R.M.; Olivera-Salguero, R.; Rodríguez-Perales, S.; Pons, T.; Setién, F.; Aiza, G.; Valdés-Mas, R.; Bertini, A.; Pineda, M.; et al. Germline Mutations in the Spindle Assembly Checkpoint Genes BUB1 and BUB3 Are Infrequent in Familial Colorectal Cancer and Polyposis. *Mol Cancer* **2018**, *17*, 23, doi:10.1186/s12943-018-0762-8.

34. Satoh, R.; Hagihara, K.; Sugiura, R. Rae1-Mediated Nuclear Export of Rnc1 Is an Important Determinant in Controlling MAPK Signaling. *Curr Genet* **2018**, *64*, 103–108, doi:10.1007/s00294-017-0732-5.

35. Fecher, L.A.; Amaravadi, R.K.; Flaherty, K.T. The MAPK Pathway in Melanoma. *Curr Opin Oncol* **2008**, *20*, 183–189, doi:10.1097/CCO.0b013e3282f5271c.

36. Burczynski, M.E.; Twine, N.C.; Dukart, G.; Marshall, B.; Hidalgo, M.; Stadler, W.M.; Logan, T.; Dutcher, J.; Hudes, G.; Trepicchio, W.L.; et al. Transcriptional Profiles in Peripheral Blood Mononuclear Cells Prognostic of Clinical Outcomes in Patients with Advanced Renal Cell Carcinoma. *Clin Cancer Res* **2005**, *11*, 1181–1189.

37. Liu, X.-S.; Genet, M.D.; Haines, J.E.; Mehanna, E.K.; Wu, S.; Chen, H.-I.H.; Chen, Y.; Qureshi, A.A.; Han, J.; Chen, X.; et al. ZBTB7A Suppresses Melanoma Metastasis by Transcriptionally Repressing MCAM. *Mol Cancer Res* **2015**, *13*, 1206–1217, doi:10.1158/1541-7786.MCR-15-0169.

38. Jen, J.; Wang, Y.-C. Zinc Finger Proteins in Cancer Progression. *J Biomed Sci* **2016**, *23*, 53, doi:10.1186/s12929-016-0269-9.

39. Constantinou, C.; Spella, M.; Chondrou, V.; Patrinos, G.P.; Papachatzopoulou, A.; Sgourou, A. The Multi-Faceted Functioning Portrait of LRF/ZBTB7A. *Hum Genomics* **2019**, *13*, 66, doi:10.1186/s40246-019-0252-0.

40. Ishida, T.; Kundu, R.K.; Yang, E.; Hirata, K.; Ho, Y.-D.; Quertermous, T. Targeted Disruption of Endothelial Cell-Selective Adhesion Molecule Inhibits Angiogenic Processes in Vitro and in Vivo. *J Biol Chem* **2003**, *278*, 34598–34604, doi:10.1074/jbc.M304890200.

41. Cangara, H.M.; Ishida, T.; Hara, T.; Sun, L.; Toh, R.; Rikitake, Y.; Kundu, R.K.; Quertermous, T.; Hirata, K.; Hayashi, Y. Role of Endothelial Cell-Selective Adhesion Molecule in Hematogeneous Metastasis. *Microvasc Res* **2010**, *80*, 133–141, doi:10.1016/j.mvr.2010.02.006.

42. Liu, Z.; Lv, Y.J.; Song, Y.P.; Li, X.H.; Du, Y.N.; Wang, C.H.; Hu, L.K. Lysosomal Membrane Protein TMEM192 Deficiency Triggers Crosstalk between Autophagy and Apoptosis in HepG2 Hepatoma Cells. *Oncol Rep* **2012**, *28*, 985–991, doi:10.3892/or.2012.1881.

43. Nguyen, T.L.; Schneppenheim, J.; Rudnik, S.; Lüllmann-Rauch, R.; Bernreuther, C.; Hermans-Borgmeyer, I.; Glatzel, M.; Saftig, P.; Schröder, B. Functional Characterization of the Lysosomal Membrane Protein TMEM192 in Mice. *Oncotarget* **2017**, *8*, 43635–43652, doi:10.18632/oncotarget.17514.

44. Kwok, W.K.; Pang, J.C.S.; Lo, K.W.; Ng, H.-K. Role of the RARRES1 Gene in Nasopharyngeal Carcinoma. *Cancer Genet Cytogenet* **2009**, *194*, 58–64, doi:10.1016/j.cancergencyto.2009.06.005.

45. Roy, A.; Ramalinga, M.; Kim, O.J.; Chijioke, J.; Lynch, S.; Byers, S.; Kumar, D. Multiple Roles of RARRES1 in Prostate Cancer: Autophagy Induction and Angiogenesis Inhibition. *PLoS One* **2017**, *12*, e0180344, doi:10.1371/journal.pone.0180344.

46. Mithani, S.K.; Smith, I.M.; Califano, J.A. Use of Integrative Epigenetic and Cytogenetic Analyses to Identify Novel Tumor-Suppressor Genes in Malignant Melanoma. *Melanoma Res* **2011**, *21*, 298–307, doi:10.1097/CMR.0b013e328344a003.

47. Shyu, R.-Y.; Wang, C.-H.; Wu, C.-C.; Chen, M.-L.; Lee, M.-C.; Wang, L.-K.; Jiang, S.-Y.; Tsai, F.-M. Tazarotene-Induced Gene 1 Enhanced Cervical Cell Autophagy through Transmembrane Protein 192. *Mol Cells* **2016**, *39*, 877–887, doi:10.14348/molcells.2016.0161.

48. Vassilopoulos, S.; Esk, C.; Hoshino, S.; Funke, B.H.; Chen, C.-Y.; Plocik, A.M.; Wright, W.E.; Kucherlapati, R.; Brodsky, F.M. A Role for the CHC22 Clathrin Heavy-Chain Isoform in Human Glucose Metabolism. *Science* **2009**, *324*, 1192–1196, doi:10.1126/science.1171529.

49. Royle, S.J. Protein Adaptation: Mitotic Functions for Membrane Trafficking Proteins. *Nat Rev Mol Cell Biol* **2013**, *14*, 592–599, doi:10.1038/nrm3641.

50. Hood, F.E.; Royle, S.J. Functional Equivalence of the Clathrin Heavy Chains CHC17 and CHC22 in Endocytosis and Mitosis. *J Cell Sci* **2009**, *122*, 2185–2190, doi:10.1242/jcs.046177.

51. Mosesson, Y.; Mills, G.B.; Yarden, Y. Derailed Endocytosis: An Emerging Feature of Cancer. *Nat Rev Cancer* **2008**, *8*, 835–850, doi:10.1038/nrc2521.

52. Davila, J.I.; Starr, J.S.; Attia, S.; Wang, C.; Knudson, R.A.; Necela, B.M.; Sarangi, V.; Sun, Z.; Ren, Y.; Casler, J.D.; et al. Comprehensive Genomic Profiling of a Rare Thyroid Follicular Dendritic Cell Sarcoma. *Rare Tumors* **2017**, *9*, 6834, doi:10.4081/rt.2017.6834.

53. Kedra, D.; Peyrard, M.; Fransson, I.; Collins, J.E.; Dunham, I.; Roe, B.A.; Dumanski, J.P. Characterization of a Second Human Clathrin Heavy Chain Polypeptide Gene (CLH-22) from Chromosome 22q11. *Hum Mol Genet* **1996**, *5*, 625–631, doi:10.1093/hmg/5.5.625.

54. Li, S.; Wang, L.; Ma, Z.; Ma, Y.; Zhao, J.; Peng, B.O.; Qiao, Z. Sequencing Study on Familial Lung Squamous Cancer. *Oncol Lett* **2015**, *10*, 2634–2638, doi:10.3892/ol.2015.3583.

55. Caballero-Díaz, D.; Bertran, E.; Peñuelas-Haro, I.; Moreno-Càceres, J.; Malfettone, A.; López-Luque, J.; Addante, A.; Herrera, B.; Sánchez, A.; Alay, A.; et al. Clathrin Switches Transforming Growth Factor-β Role to pro-Tumorigenic in Liver Cancer. *J Hepatol* **2020**, *72*, 125–134, doi:10.1016/j.jhep.2019.09.012.

56. Ohmori, K.; Endo, Y.; Yoshida, Y.; Ohata, H.; Taya, Y.; Enari, M. Monomeric but Not Trimeric Clathrin Heavy Chain Regulates P53-Mediated Transcription. *Oncogene* **2008**, *27*, 2215–2227, doi:10.1038/sj.onc.1210854.

57. Ybe, J.A. Novel Clathrin Activity: Developments in Health and Disease. *Biomol Concepts* **2014**, *5*, 175–182, doi:10.1515/bmc-2013-0040.

58. Argani, P.; Lui, M.Y.; Couturier, J.; Bouvier, R.; Fournet, J.-C.; Ladanyi, M. A Novel CLTC-TFE3 Gene Fusion in Pediatric Renal Adenocarcinoma with t(X;17)(P11.2;Q23). *Oncogene* **2003**, *22*, 5374–5378, doi:10.1038/sj.onc.1206686.

59. Poli, J.; Gasser, S.M.; Papamichos-Chronakis, M. The INO80 Remodeller in Transcription, Replication and Repair. *Philos Trans R Soc Lond B Biol Sci* **2017**, *372*, doi:10.1098/rstb.2016.0290.

60. Zhou, B.; Wang, L.; Zhang, S.; Bennett, B.D.; He, F.; Zhang, Y.; Xiong, C.; Han, L.; Diao, L.; Li, P.; et al. INO80 Governs Superenhancer-Mediated Oncogenic Transcription and Tumor Growth in Melanoma. *Genes Dev* **2016**, *30*, 1440–1453, doi:10.1101/gad.277178.115.

61. Ricketts, C.J.; Crooks, D.R.; Sourbier, C.; Schmidt, L.S.; Srinivasan, R.; Linehan, W.M. SnapShot: Renal Cell Carcinoma. *Cancer Cell* **2016**, *29*, 610-610.e1, doi:10.1016/j.ccell.2016.03.021.

62. Beckwith, S.L.; Schwartz, E.K.; García-Nieto, P.E.; King, D.A.; Gowans, G.J.; Wong, K.M.; Eckley, T.L.; Paraschuk, A.P.; Peltan, E.L.; Lee, L.R.; et al. The INO80 Chromatin Remodeler Sustains Metabolic Stability by Promoting TOR Signaling and Regulating Histone Acetylation. *PLoS Genet* **2018**, *14*, e1007216, doi:10.1371/journal.pgen.1007216.

63. Wiesner, T.; Obenauf, A.C.; Murali, R.; Fried, I.; Griewank, K.G.; Ulz, P.; Windpassinger, C.; Wackernagel, W.; Loy, S.; Wolf, I.; et al. Germline Mutations in BAP1 Predispose to Melanocytic Tumors. *Nat Genet* **2011**, *43*, 1018–1021, doi:10.1038/ng.910.

64. Peña-Llopis, S.; Vega-Rubín-de-Celis, S.; Liao, A.; Leng, N.; Pavía-Jiménez, A.; Wang, S.; Yamasaki, T.; Zhrebker, L.; Sivanand, S.; Spence, P.; et al. BAP1 Loss Defines a New Class of Renal Cell Carcinoma. *Nat Genet* **2012**, *44*, 751–759, doi:10.1038/ng.2323.

65. Lee, H.-S.; Lee, S.-A.; Hur, S.-K.; Seo, J.-W.; Kwon, J. Stabilization and Targeting of INO80 to Replication Forks by BAP1 during Normal DNA Synthesis. *Nat Commun* **2014**, *5*, 5128, doi:10.1038/ncomms6128.

66. Sanchez-Pulido, L.; Kong, L.; Ponting, C.P. A Common Ancestry for BAP1 and Uch37 Regulators. *Bioinformatics* **2012**, *28*, 1953–1956, doi:10.1093/bioinformatics/bts319.

67. Vander Linden, R.T.; Hemmis, C.W.; Schmitt, B.; Ndoja, A.; Whitby, F.G.; Robinson, H.; Cohen, R.E.; Yao, T.; Hill, C.P. Structural Basis for the Activation and Inhibition of the UCH37 Deubiquitylase. *Mol Cell* **2015**, *57*, 901–911, doi:10.1016/j.molcel.2015.01.016.

68. Iyer, N.G.; Ozdag, H.; Caldas, C. P300/CBP and Cancer. *Oncogene* **2004**, *23*, 4225–4231, doi:10.1038/sj.onc.1207118.

69. Attar, N.; Kurdistani, S.K. Exploitation of EP300 and CREBBP Lysine Acetyltransferases by Cancer. *Cold Spring Harb Perspect Med* **2017**, *7*, doi:10.1101/cshperspect.a026534.

70. Gutiérrez-Salmerón, M.; García-Martínez, J.M.; Martínez-Useros, J.; Fernández-Aceñero, M.J.; Viollet, B.; Olivier, S.; Chauhan, J.; Lucena, S.R.; De la Vieja, A.; Goding, C.R.; et al. Paradoxical Activation of AMPK by Glucose Drives Selective EP300 Activity in Colorectal Cancer. *PLoS Biol* **2020**, *18*, e3000732, doi:10.1371/journal.pbio.3000732.

71. Yuan, Z.M.; Huang, Y.; Ishiko, T.; Nakada, S.; Utsugisawa, T.; Shioya, H.; Utsugisawa, Y.; Yokoyama, K.; Weichselbaum, R.; Shi, Y.; et al. Role for P300 in Stabilization of P53 in the Response to DNA Damage. *J Biol Chem* **1999**, *274*, 1883–1886, doi:10.1074/jbc.274.4.1883.

72. Zoni, E.; van der Pluijm, G.; Gray, P.C.; Kruithof-de Julio, M. Epithelial Plasticity in Cancer: Unmasking a MicroRNA Network for TGF-β-, Notch-, and Wnt-Mediated EMT. *J Oncol* **2015**, *2015*, 198967, doi:10.1155/2015/198967.

73. Avantaggiati, M.L.; Ogryzko, V.; Gardner, K.; Giordano, A.; Levine, A.S.; Kelly, K. Recruitment of P300/CBP in P53-Dependent Signal Pathways. *Cell* **1997**, *89*, 1175–1184, doi:10.1016/s0092-8674(00)80304-9.

74. Bhandaru, M.; Ardekani, G.S.; Zhang, G.; Martinka, M.; McElwee, K.J.; Li, G.; Rotte, A. A Combination of P300 and Braf Expression in the Diagnosis and Prognosis of Melanoma. *BMC Cancer* **2014**, *14*, 398, doi:10.1186/1471-2407-14-398.

75. Semenza, G.L. Hypoxia-Inducible Factor 1 (HIF-1) Pathway. *Sci STKE* **2007**, *2007*, cm8, doi:10.1126/stke.4072007cm8.

76. Wang, R.; He, Y.; Robinson, V.; Yang, Z.; Hessler, P.; Lasko, L.M.; Lu, X.; Bhathena, A.; Lai, A.; Uziel, T.; et al. Targeting Lineage-Specific MITF Pathway in Human Melanoma Cell Lines by A-485, the Selective Small-Molecule Inhibitor of P300/CBP. *Mol Cancer Ther* **2018**, *17*, 2543–2550, doi:10.1158/1535-7163.MCT-18-0511.

77. Wohlrab, C.; Vissers, M.C.M.; Phillips, E.; Morrin, H.; Robinson, B.A.; Dachs, G.U. The Association Between Ascorbate and the Hypoxia-Inducible Factors in Human Renal Cell Carcinoma Requires a Functional Von Hippel-Lindau Protein. *Front Oncol* **2018**, *8*, 574, doi:10.3389/fonc.2018.00574.

78. Wang, X.-T.; Fang, R.; Ye, S.-B.; Zhang, R.-S.; Li, R.; Wang, X.; Ji, R.-H.; Lu, Z.-F.; Ma, H.-H.; Zhou, X.-J.; et al. Targeted Next-Generation Sequencing Revealed Distinct Clinicopathologic and Molecular Features of VCL-ALK RCC: A Unique Case from an Older Patient without Clinical Evidence of Sickle Cell Trait. *Pathol Res Pract* **2019**, *215*, 152651, doi:10.1016/j.prp.2019.152651.

79. Rothhammer, T.; Bosserhoff, A.-K. Epigenetic Events in Malignant Melanoma. *Pigment Cell Res* **2007**, *20*, 92–111, doi:10.1111/j.1600-0749.2007.00367.x.

80. Yao, X.; Tan, J.; Lim, K.J.; Koh, J.; Ooi, W.F.; Li, Z.; Huang, D.; Xing, M.; Chan, Y.S.; Qu, J.Z.; et al. VHL Deficiency Drives Enhancer Activation of Oncogenes in Clear Cell Renal Cell Carcinoma. *Cancer Discov* **2017**, *7*, 1284–1305, doi:10.1158/2159-8290.CD-17-0375.

81. Lee, S.-H.; Kang, J.H.; Ha, J.S.; Lee, J.-S.; Oh, S.-J.; Choi, H.-J.; Song, J.; Kim, S.-Y. Transglutaminase 2-Mediated P53 Depletion Promotes Angiogenesis by Increasing HIF-1α-P300 Binding in Renal Cell Carcinoma. *Int J Mol Sci* **2020**, *21*, doi:10.3390/ijms21145042.

82. Lee, Y.-G.; Macoska, J.A.; Korenchuk, S.; Pienta, K.J. MIM, a Potential Metastasis Suppressor Gene in Bladder Cancer. *Neoplasia* **2002**, *4*, 291–294, doi:10.1038/sj.neo.7900231.

83. Alazami, A.M.; Patel, N.; Shamseldin, H.E.; Anazi, S.; Al-Dosari, M.S.; Alzahrani, F.; Hijazi, H.; Alshammari, M.; Aldahmesh, M.A.; Salih, M.A.; et al. Accelerating Novel Candidate Gene Discovery in Neurogenetic Disorders via Whole-Exome Sequencing of Prescreened Multiplex Consanguineous Families. *Cell Rep* **2015**, *10*, 148–161, doi:10.1016/j.celrep.2014.12.015.

84. Chatzi, C.; Zhang, Y.; Hendricks, W.D.; Chen, Y.; Schnell, E.; Goodman, R.H.; Westbrook, G.L. Exercise-Induced Enhancement of Synaptic Function Triggered by the Inverse BAR Protein, Mtss1L. *Elife* **2019**, *8*, doi:10.7554/eLife.45920.

85. Mertz, K.D.; Pathria, G.; Wagner, C.; Saarikangas, J.; Sboner, A.; Romanov, J.; Gschaider, M.; Lenz, F.; Neumann, F.; Schreiner, W.; et al. MTSS1 Is a Metastasis Driver in a Subset of Human Melanomas. *Nat Commun* **2014**, *5*, 3465, doi:10.1038/ncomms4465.

86. Callahan, C.A.; Ofstad, T.; Horng, L.; Wang, J.K.; Zhen, H.H.; Coulombe, P.A.; Oro, A.E. MIM/BEG4, a Sonic Hedgehog-Responsive Gene That Potentiates Gli-Dependent Transcription. *Genes Dev* **2004**, *18*, 2724–2729, doi:10.1101/gad.1221804.

87. Epstein, E.H. Basal Cell Carcinomas: Attack of the Hedgehog. *Nat Rev Cancer* **2008**, *8*, 743–754, doi:10.1038/nrc2503.

88. Yu, D.; Zhan, X.H.; Zhao, X.F.; Williams, M.S.; Carey, G.B.; Smith, E.; Scott, D.; Zhu, J.; Guo, Y.; Cherukuri, S.; et al. Mice Deficient in MIM Expression Are Predisposed to Lymphomagenesis. *Oncogene* **2012**, *31*, 3561–3568, doi:10.1038/onc.2011.509.

89. Li, N.; Huang, D.; Lu, N.; Luo, L. Role of the LKB1/AMPK Pathway in Tumor Invasion and Metastasis of Cancer Cells (Review). *Oncol Rep* **2015**, *34*, 2821–2826, doi:10.3892/or.2015.4288.

90. Dalgliesh, G.L.; Furge, K.; Greenman, C.; Chen, L.; Bignell, G.; Butler, A.; Davies, H.; Edkins, S.; Hardy, C.; Latimer, C.; et al. Systematic Sequencing of Renal Carcinoma Reveals Inactivation of Histone Modifying Genes. *Nature* **2010**, *463*, 360–363, doi:10.1038/nature08672.

91. Lee, J.J.; Sholl, L.M.; Lindeman, N.I.; Granter, S.R.; Laga, A.C.; Shivdasani, P.; Chin, G.; Luke, J.J.; Ott, P.A.; Hodi, F.S.; et al. Targeted Next-Generation Sequencing Reveals High Frequency of Mutations in Epigenetic Regulators across Treatment-Naïve Patient Melanomas. *Clin Epigenetics* **2015**, *7*, 59, doi:10.1186/s13148-015-0091-3.

92. Li, L.; Miao, W.; Huang, M.; Williams, P.; Wang, Y. Integrated Genomic and Proteomic Analyses Reveal Novel Mechanisms of the Methyltransferase SETD2 in Renal Cell Carcinoma Development. *Mol Cell Proteomics* **2019**, *18*, 437–447, doi:10.1074/mcp.RA118.000957.

93. Terakawa, T.; Bisht, S.; Eeftens, J.M.; Dekker, C.; Haering, C.H.; Greene, E.C. The Condensin Complex Is a Mechanochemical Motor That Translocates along DNA. *Science* **2017**, *358*, 672–676, doi:10.1126/science.aan6516.

94. Zhang, T.; Si-Hoe, S.L.; Hudson, D.F.; Surana, U. Condensin Recruitment to Chromatin Is Inhibited by Chk2 Kinase in Response to DNA Damage. *Cell Cycle* **2016**, *15*, 3454–3470, doi:10.1080/15384101.2016.1249075.

95. Stolz, A.; Ertych, N.; Bastians, H. Tumor Suppressor CHK2: Regulator of DNA Damage Response and Mediator of Chromosomal Stability. *Clin Cancer Res* **2011**, *17*, 401–405, doi:10.1158/1078-0432.CCR-10-1215.

96. Dávalos, V.; Súarez-López, L.; Castaño, J.; Messent, A.; Abasolo, I.; Fernandez, Y.; Guerra-Moreno, A.; Espín, E.; Armengol, M.; Musulen, E.; et al. Human SMC2 Protein, a Core Subunit of Human Condensin Complex, Is a Novel Transcriptional Target of the WNT Signaling Pathway and a New Therapeutic Target. *J Biol Chem* **2012**, *287*, 43472–43481, doi:10.1074/jbc.M112.428466.

97. Strunnikov, A.V. One-Hit Wonders of Genomic Instability. *Cell Div* **2010**, *5*, 15, doi:10.1186/1747-1028-5-15.

98. Je, E.M.; Yoo, N.J.; Lee, S.H. Mutational and Expressional Analysis of SMC2 Gene in Gastric and Colorectal Cancers with Microsatellite Instability. *APMIS* **2014**, *122*, 499–504, doi:10.1111/apm.12193.

99. Feng, Y.; Liu, H.; Duan, B.; Liu, Z.; Abbruzzese, J.; Walsh, K.M.; Zhang, X.; Wei, Q. Potential Functional Variants in SMC2 and TP53 in the AURORA Pathway Genes and Risk of Pancreatic Cancer. *Carcinogenesis* **2019**, *40*, 521–528, doi:10.1093/carcin/bgz029.

100. Liao, D. Emerging Roles of the EBF Family of Transcription Factors in Tumor Suppression. *Mol Cancer Res* **2009**, *7*, 1893–1901, doi:10.1158/1541-7786.MCR-09-0229.

101. Chatterjee, A.; Stockwell, P.A.; Ahn, A.; Rodger, E.J.; Leichter, A.L.; Eccles, M.R. Genome-Wide Methylation Sequencing of Paired Primary and Metastatic Cell Lines Identifies Common DNA Methylation Changes and a Role for EBF3 as a Candidate Epigenetic Driver of Melanoma Metastasis. *Oncotarget* **2017**, *8*, 6085–6101, doi:10.18632/oncotarget.14042.

102. Artomov, M.; Stratigos, A.J.; Kim, I.; Kumar, R.; Lauss, M.; Reddy, B.Y.; Miao, B.; Daniela Robles-Espinoza, C.; Sankar, A.; Njauw, C.-N.; et al. Rare Variant, Gene-Based Association Study of Hereditary Melanoma Using Whole-Exome Sequencing. *J Natl Cancer Inst* **2017**, *109*, doi:10.1093/jnci/djx083.

103. Li, W.; Liu, J.; Zhang, B.; Bie, Q.; Qian, H.; Xu, W. Transcriptome Analysis Reveals Key Genes and Pathways Associated with Metastasis in Breast Cancer. *Onco Targets Ther* **2020**, *13*, 323–335, doi:10.2147/OTT.S226770.
