# Supplementary material for: The PI3K/mTOR Pathway Is Targeted by Rare Germline Variants in Patients with Both Melanoma and Renal Cell Carcinoma
Source: Cancers (Basel). 2021 May 7;13(9):2243. doi: 10.3390/cancers13092243 (PMC8125037; doi:10.3390/cancers13092243)
Supplement: Supplementary file 1 [file cancers-13-02243-s001.zip › Hubert_Suybeng_MelanomaRCC_Supp_File2_R2.pdf]

PIK3CD

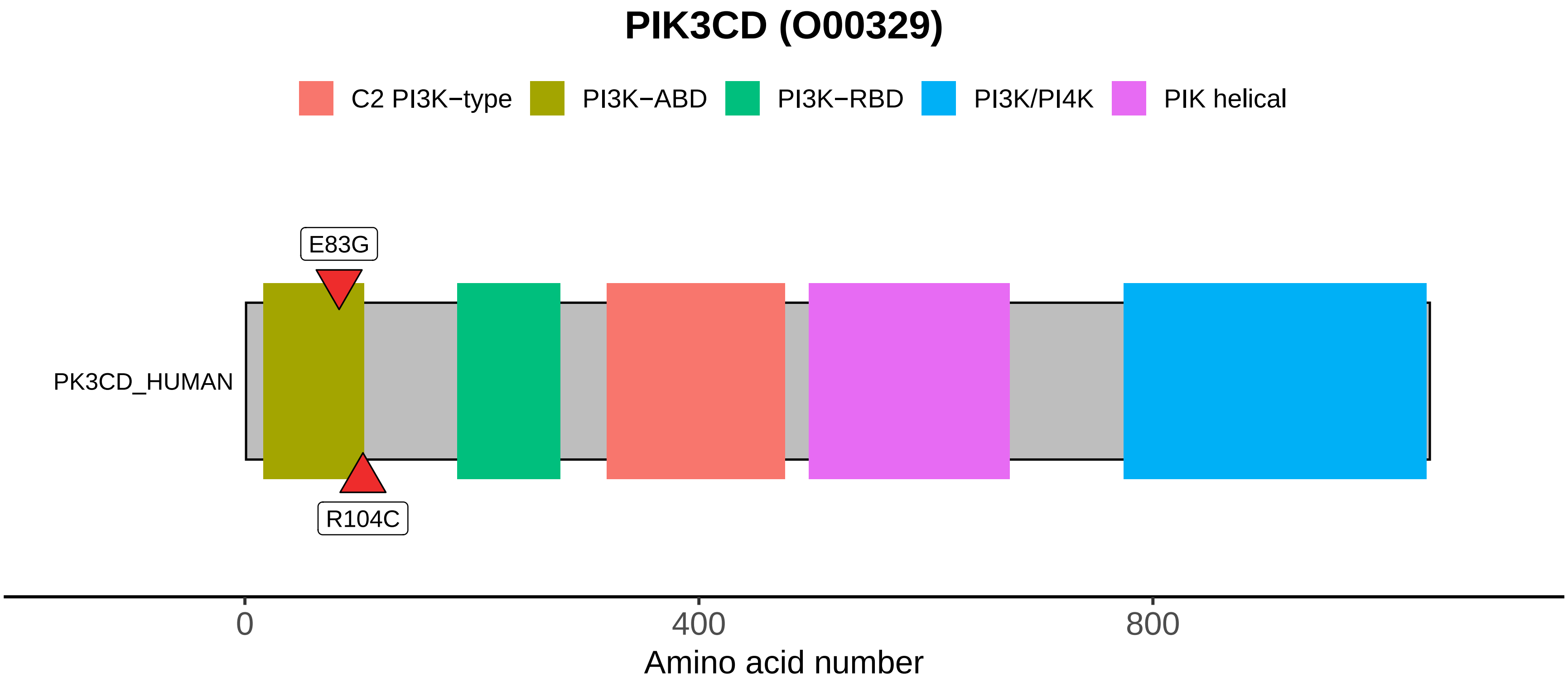

PIK3CD (O00329)
C2 PI3K−type
PI3K−ABD
PI3K−RBD
PI3K/PI4K
PIK helical
E83G
PK3CD_HUMAN
R104C
0
400
800
Amino acid number

Ribbon diagram of the p110δ-iSH2 heterodimer, including a zoomed view on the affected residues (E83G and R104C).

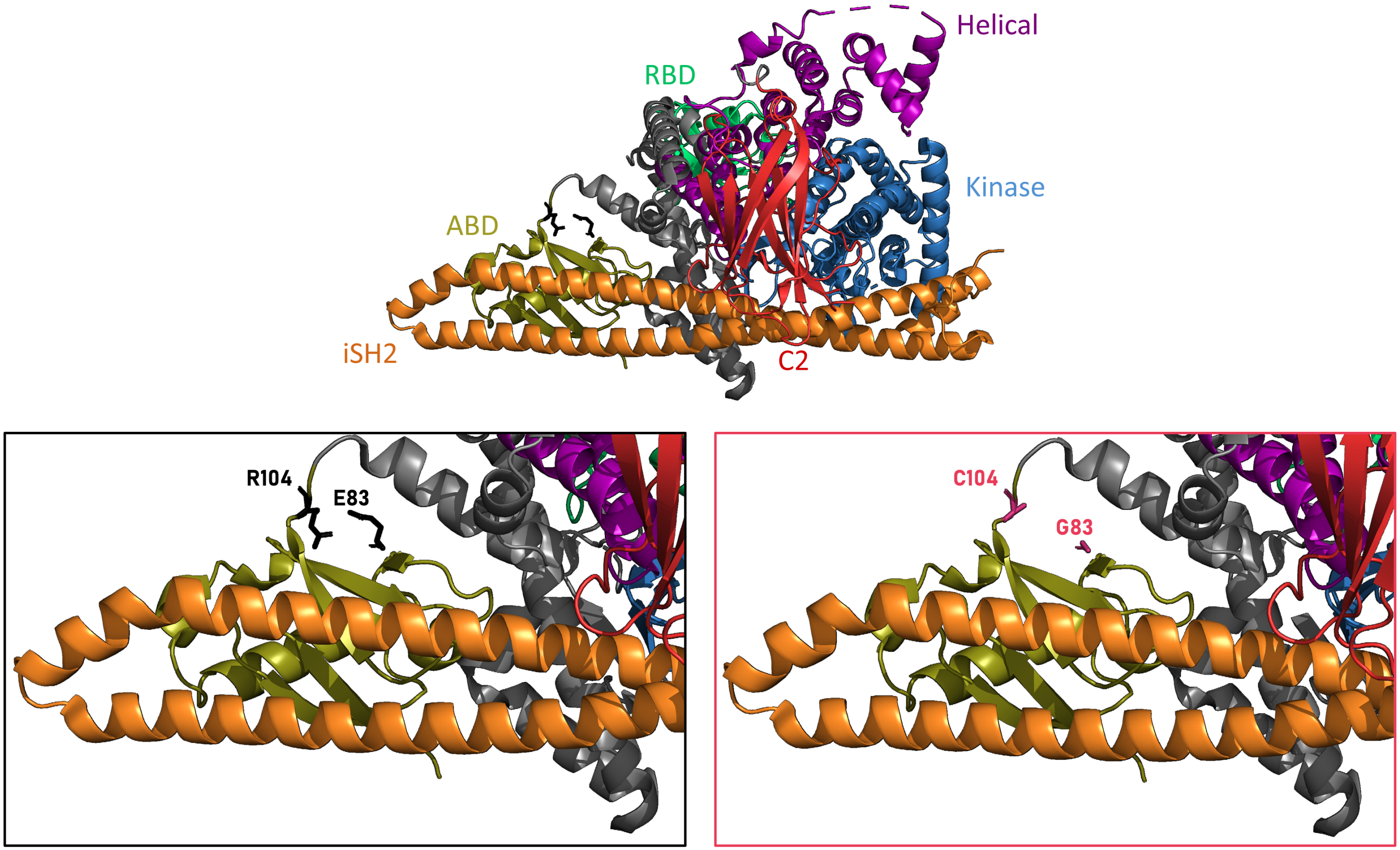

MTOR

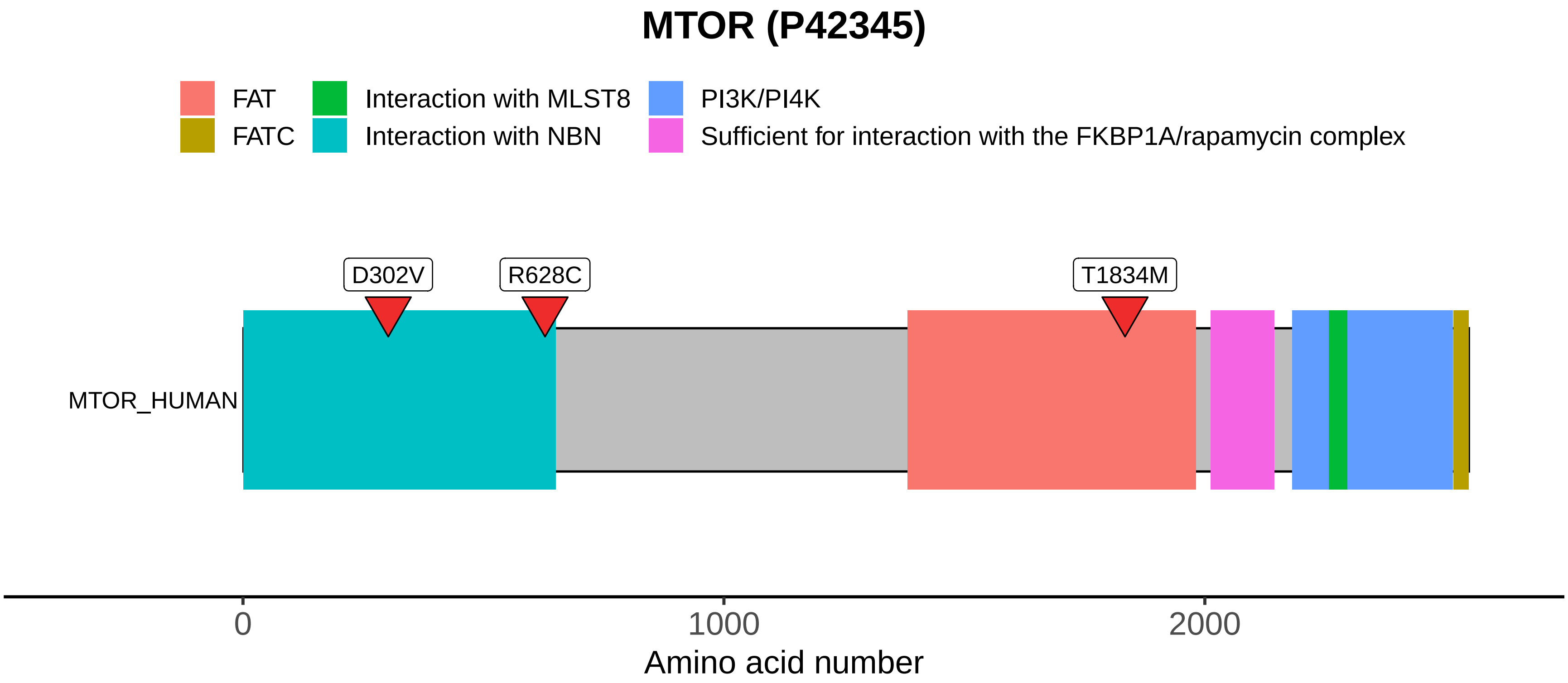

MTOR (P42345)
FAT
FATC
Interaction with MLST8
Interaction with NBN
PI3K/PI4K
Sufficient for interaction with the FKBP1A/rapamycin complex
D302V
R628C
T1834M
MTOR_HUMAN
0
1000
2000
Amino acid number

RAE1

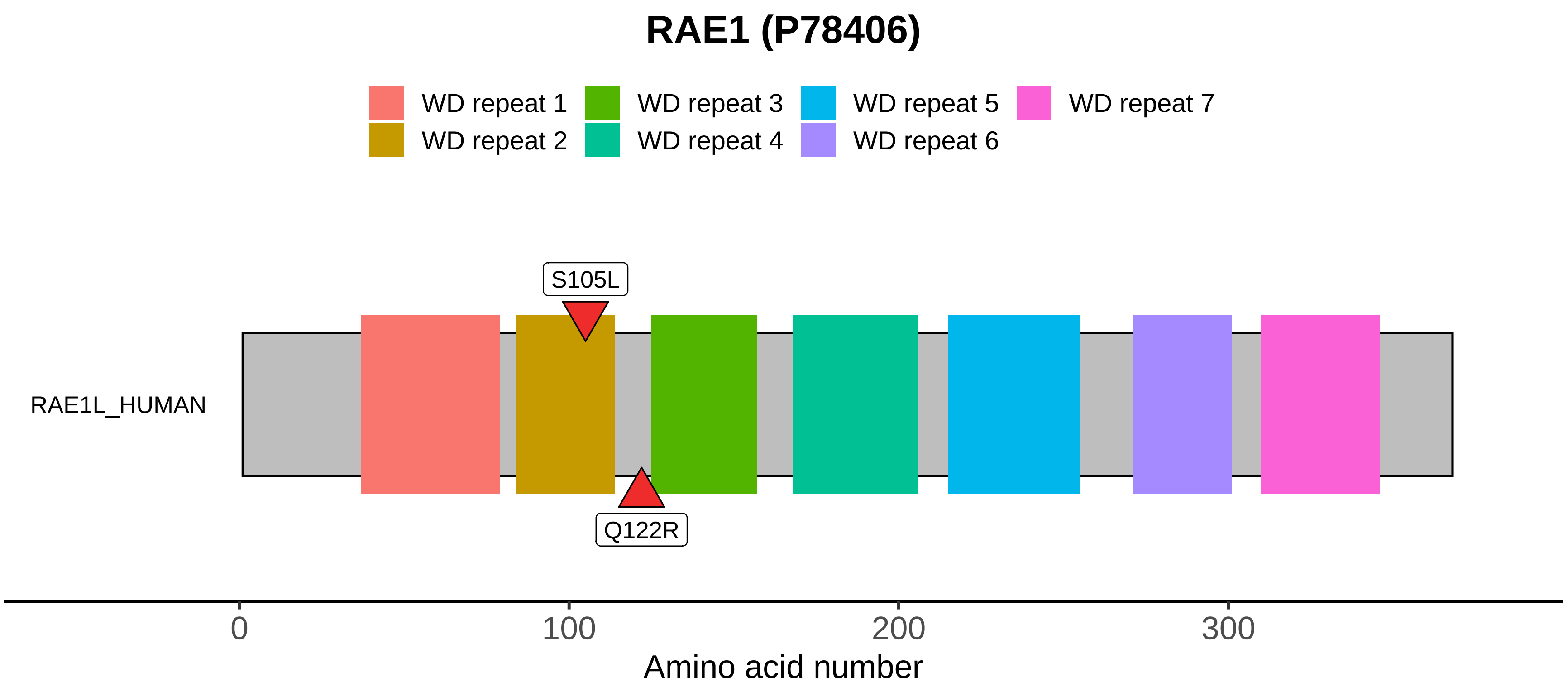
RAE1 (P78406)
WD repeat 1
WD repeat 2
WD repeat 3
WD repeat 4
WD repeat 5
WD repeat 6
WD repeat 7
S105L
Q122R
RAE1L_HUMAN
0
100
200
300
Amino acid number

ZBTB21

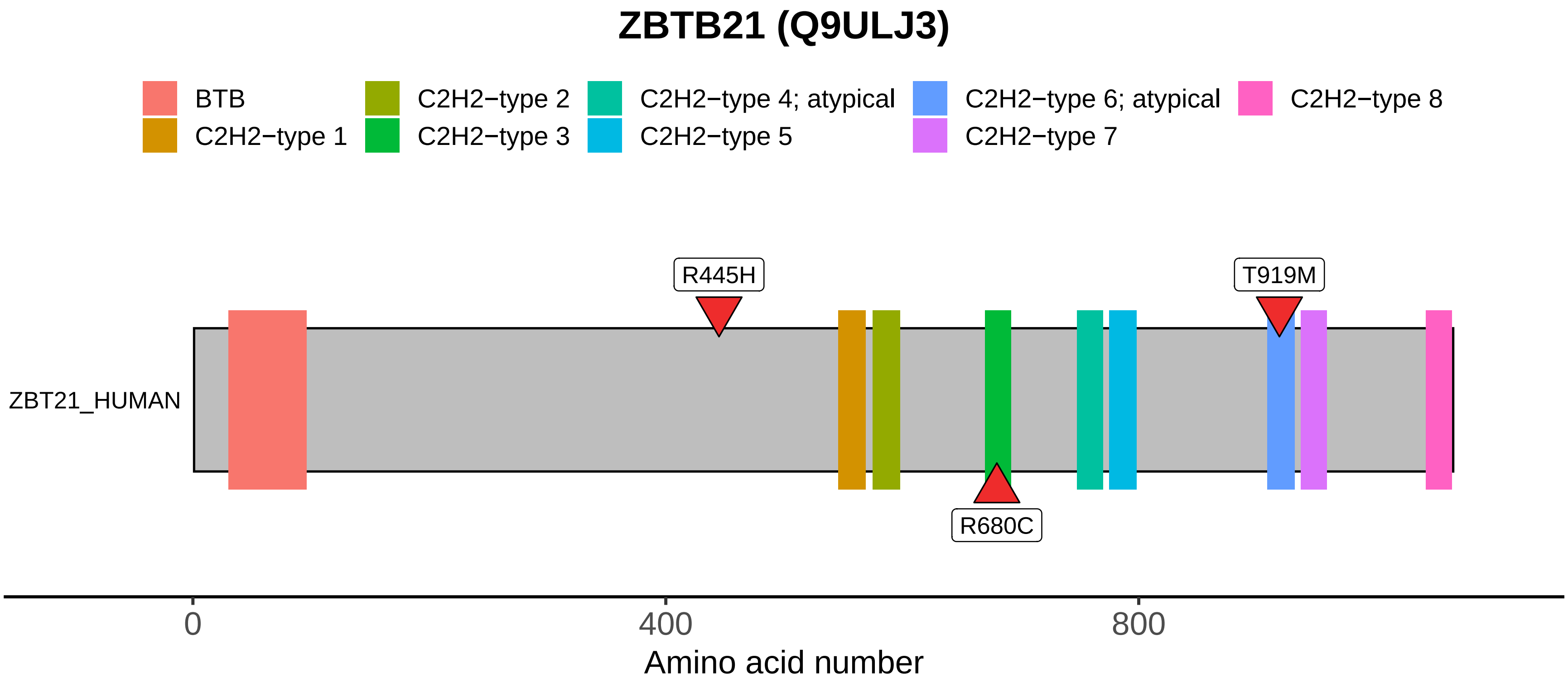

ZBTB21 (Q9ULJ3)
BTB
C2H2−type 1
C2H2−type 2
C2H2−type 3
C2H2−type 4; atypical
C2H2−type 5
C2H2−type 6; atypical
C2H2−type 7
C2H2−type 8
R445H
T919M
R680C
ZBT21_HUMAN
0
400
800
Amino acid number

ESAM

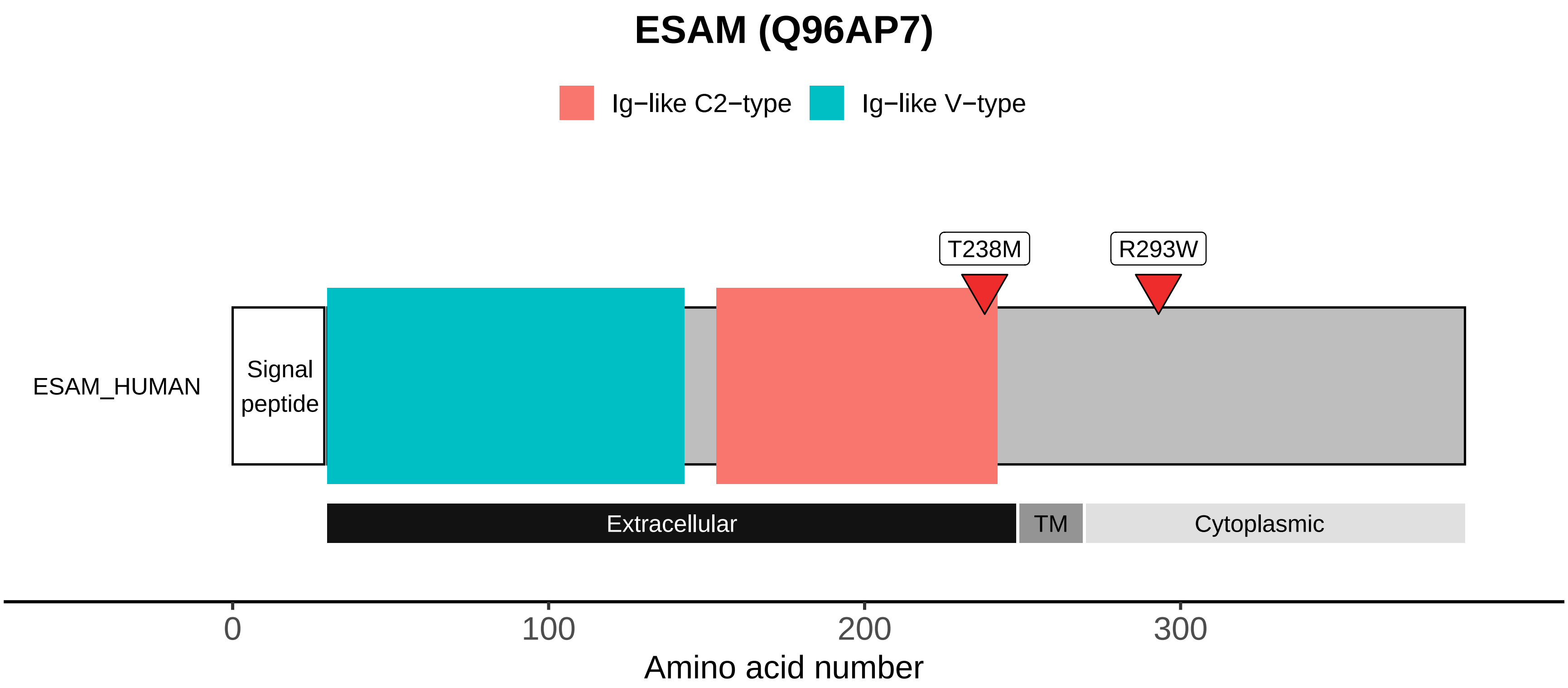

ESAM (Q96AP7)
Ig−like C2−type
Ig−like V−type
T238M
R293W
ESAM_HUMAN
Signal peptide
Extracellular
TM
Cytoplasmic
0
100
200
300
Amino acid number

TMEM192

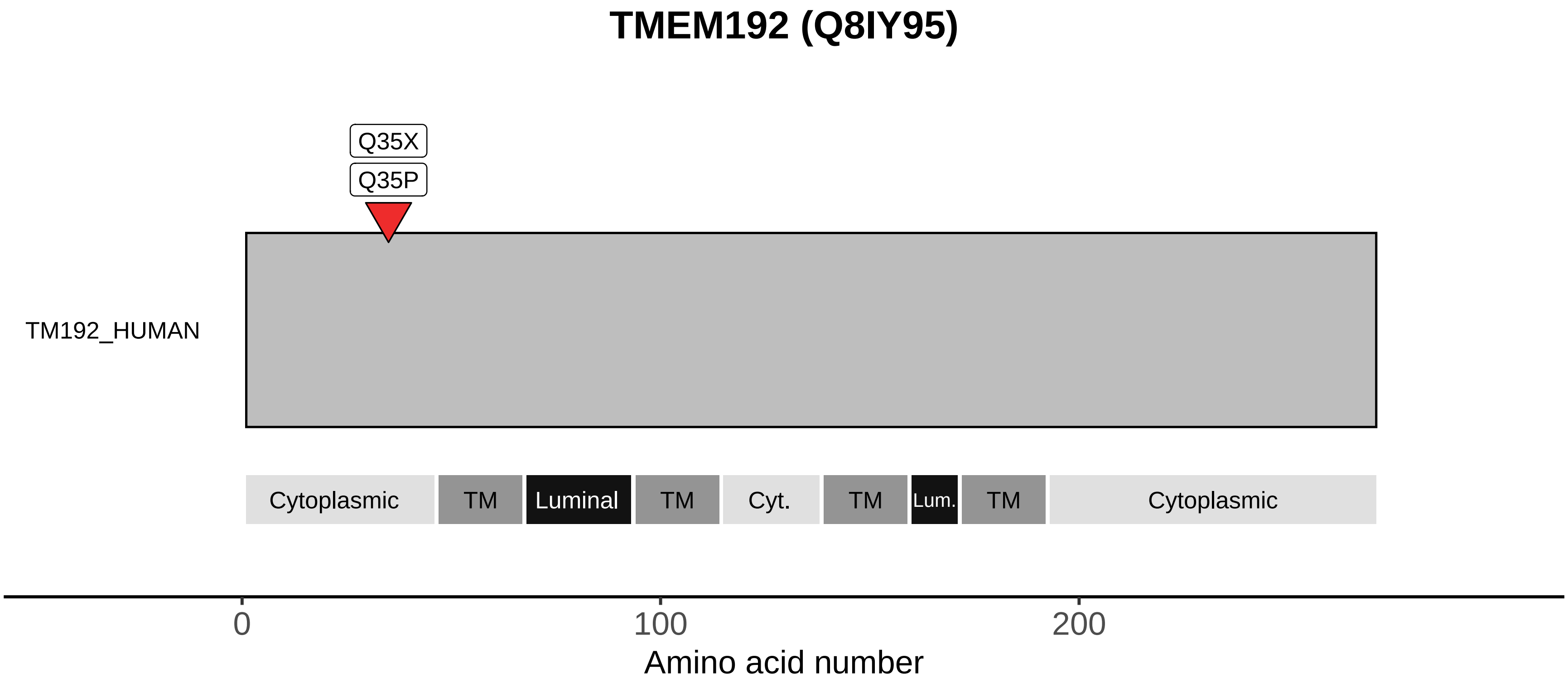
TMEM192 (Q8IY95)
Q35X
Q35P
TM192_HUMAN
Cytoplasmic
TM
Luminal
TM
Cyt.
TM
Lum.
TM
Cytoplasmic
0
100
200
Amino acid number

CLTCL1

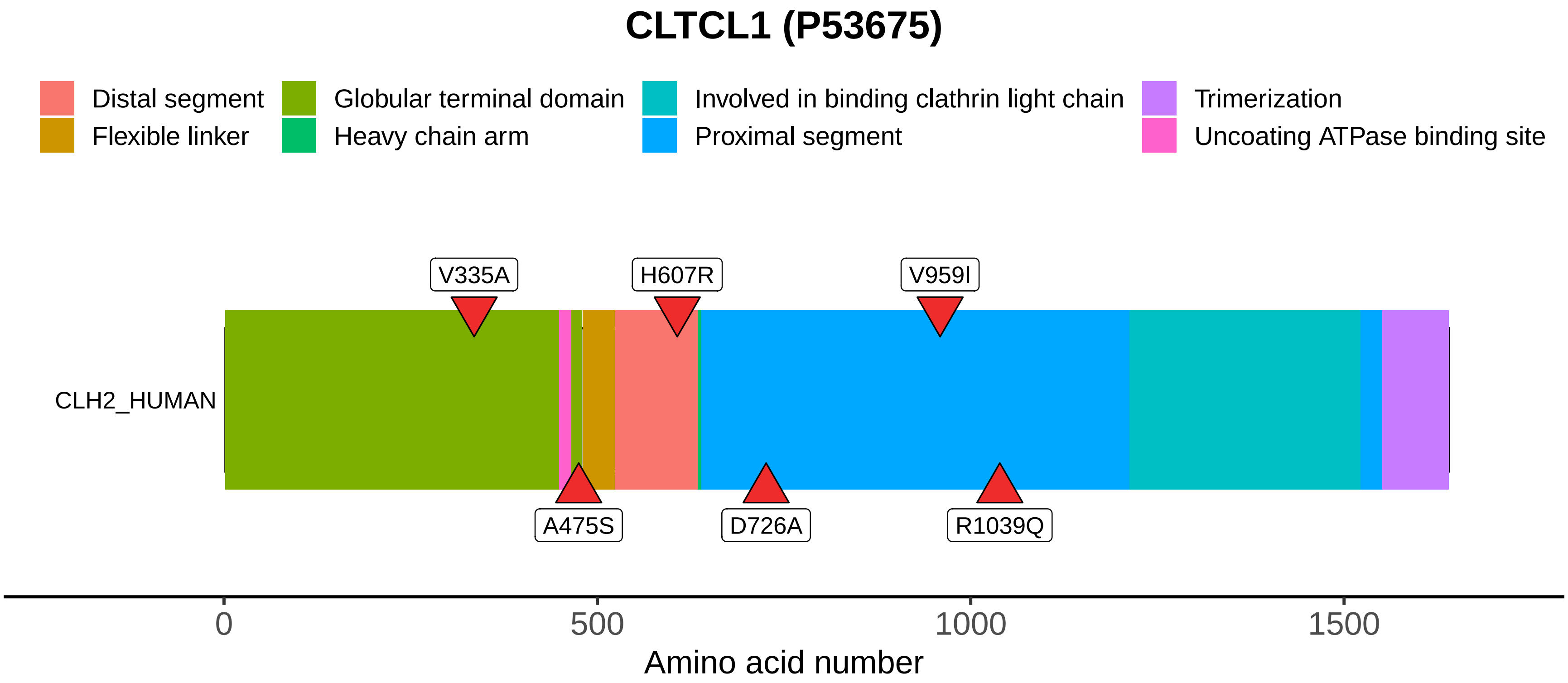

CLTCL1 (P53675)
Distal segment
Flexible linker
Globular terminal domain
Heavy chain arm
Involved in binding clathrin light chain
Proximal segment
Trimerization
Uncoating ATPase binding site
V335A
H607R
V959I
CLH2_HUMAN
A475S
D726A
R1039Q
0
500
1000
1500
Amino acid number

NFRKB

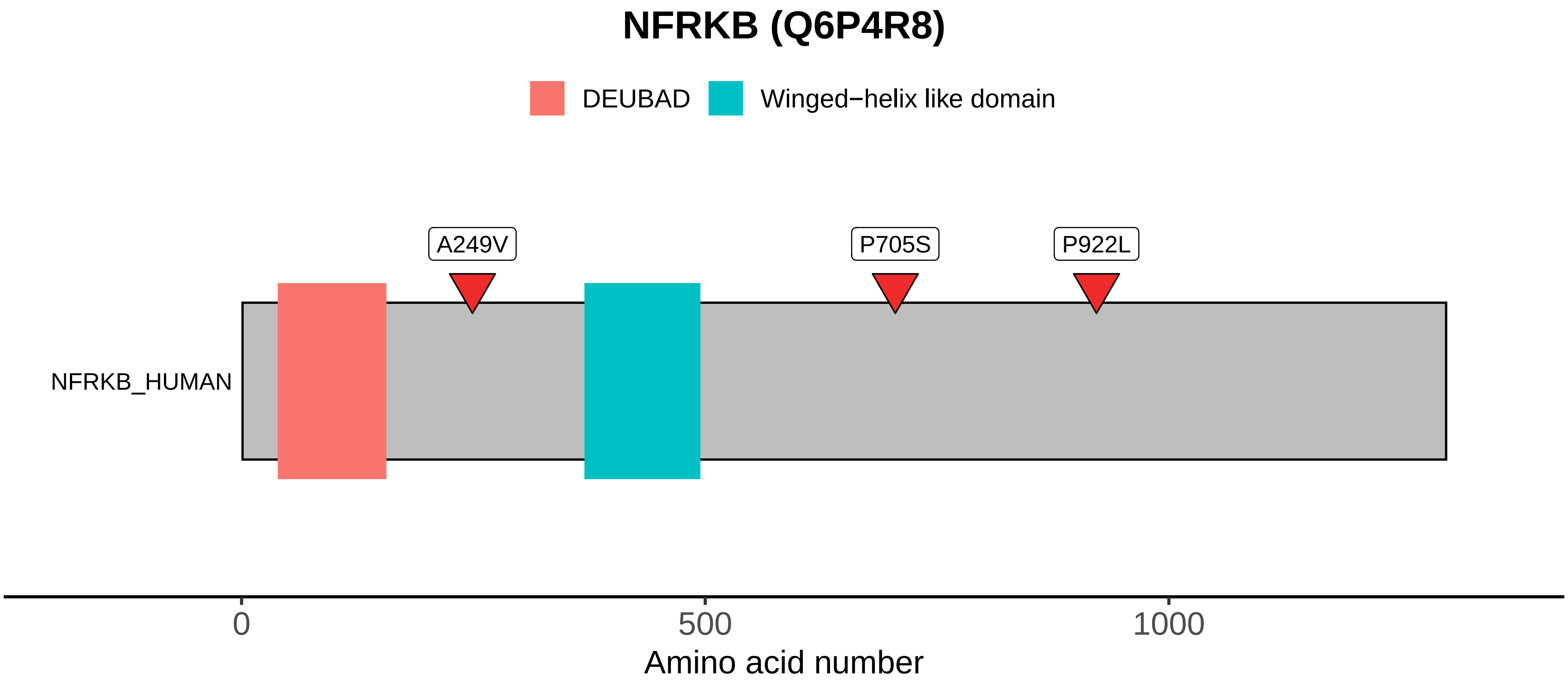
NFRKB (Q6P4R8)
DEUBAD
Winged−helix like domain
A249V
P705S
P922L
NFRKB_HUMAN
0
500
1000
Amino acid number

EP300

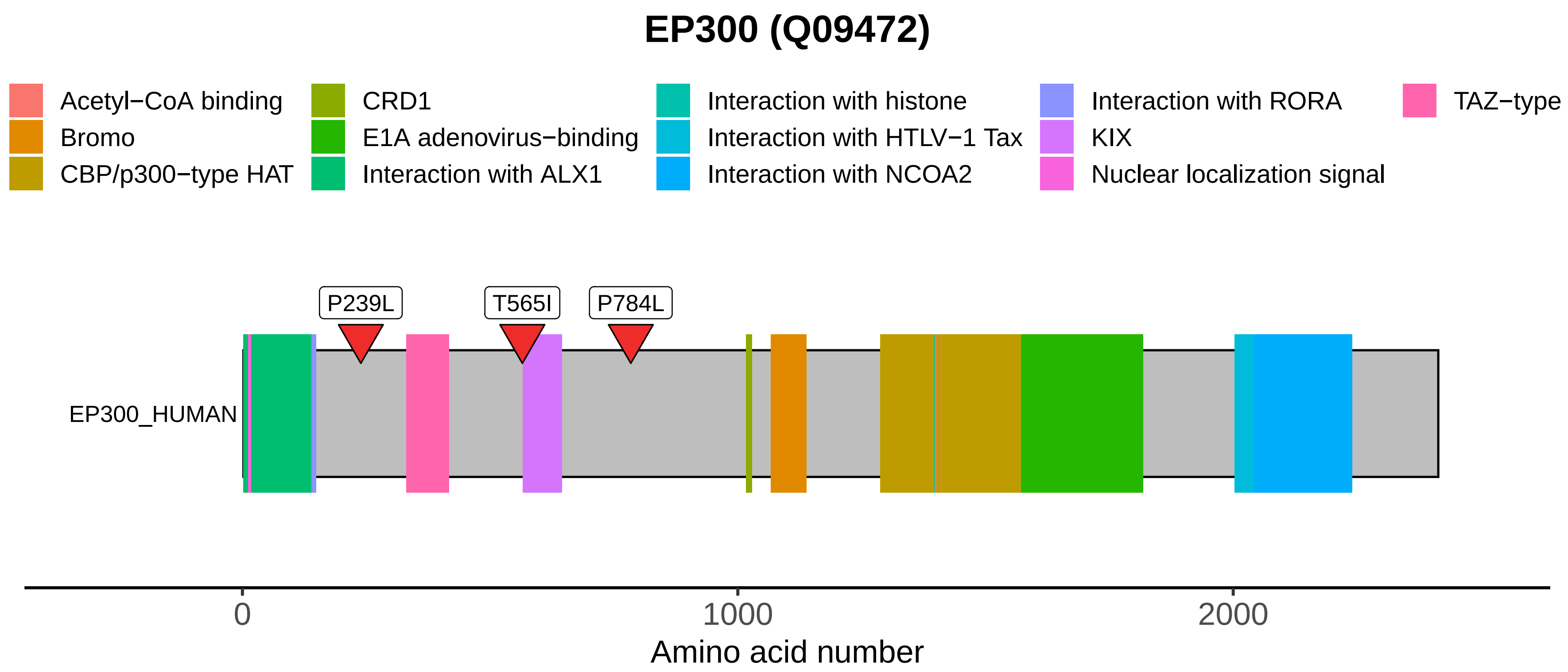

EP300 (Q09472)
Acetyl−CoA binding
Bromo
CBP/p300−type HAT
CRD1
E1A adenovirus−binding
Interaction with ALX1
Interaction with histone
Interaction with HTLV−1 Tax
Interaction with NCOA2
Interaction with RORA
KIX
Nuclear localization signal
TAZ−type
P239L
T565I
P784L
EP300_HUMAN
0
1000
2000
Amino acid number

MTSS2

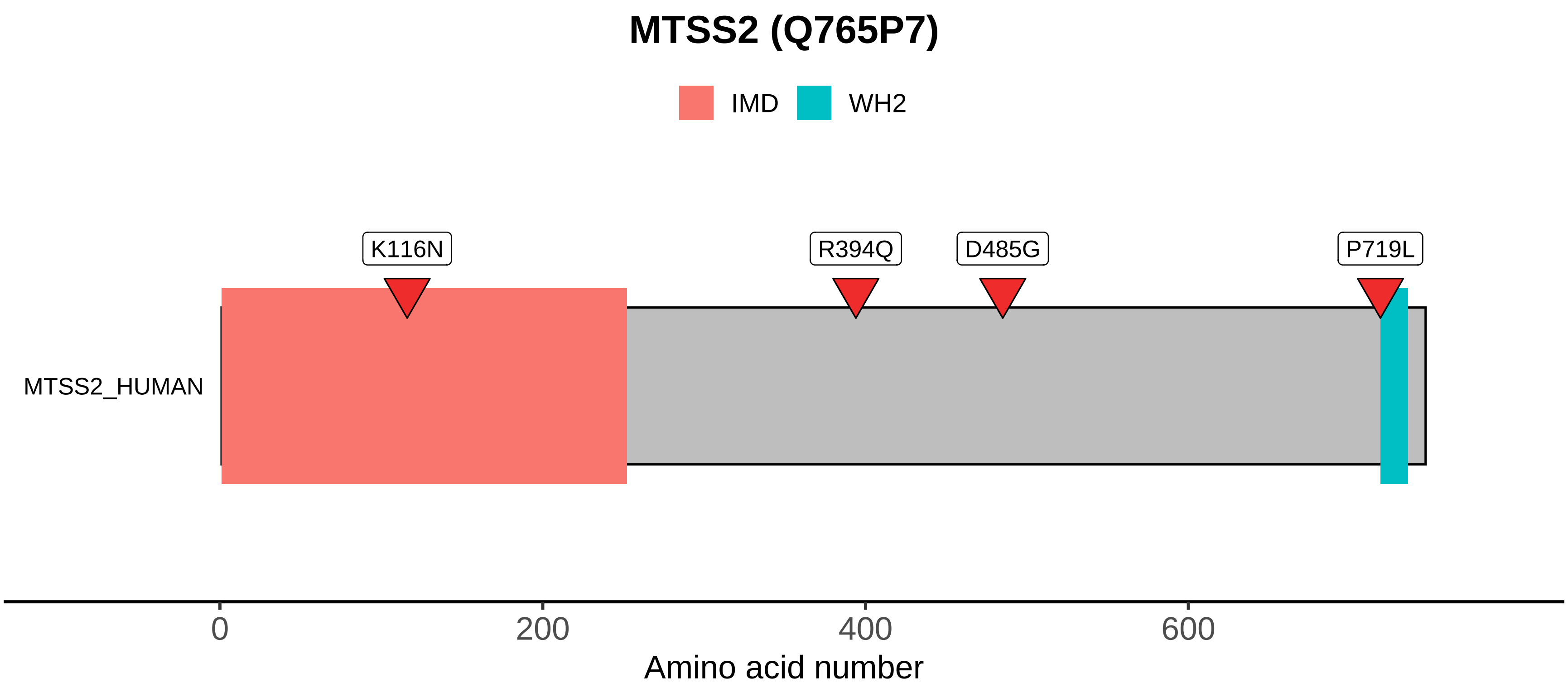

MTSS2 (Q765P7)
IMD
WH2
K116N
R394Q
D485G
P719L
MTSS2_HUMAN
0
200
400
600
Amino acid number

SETD2

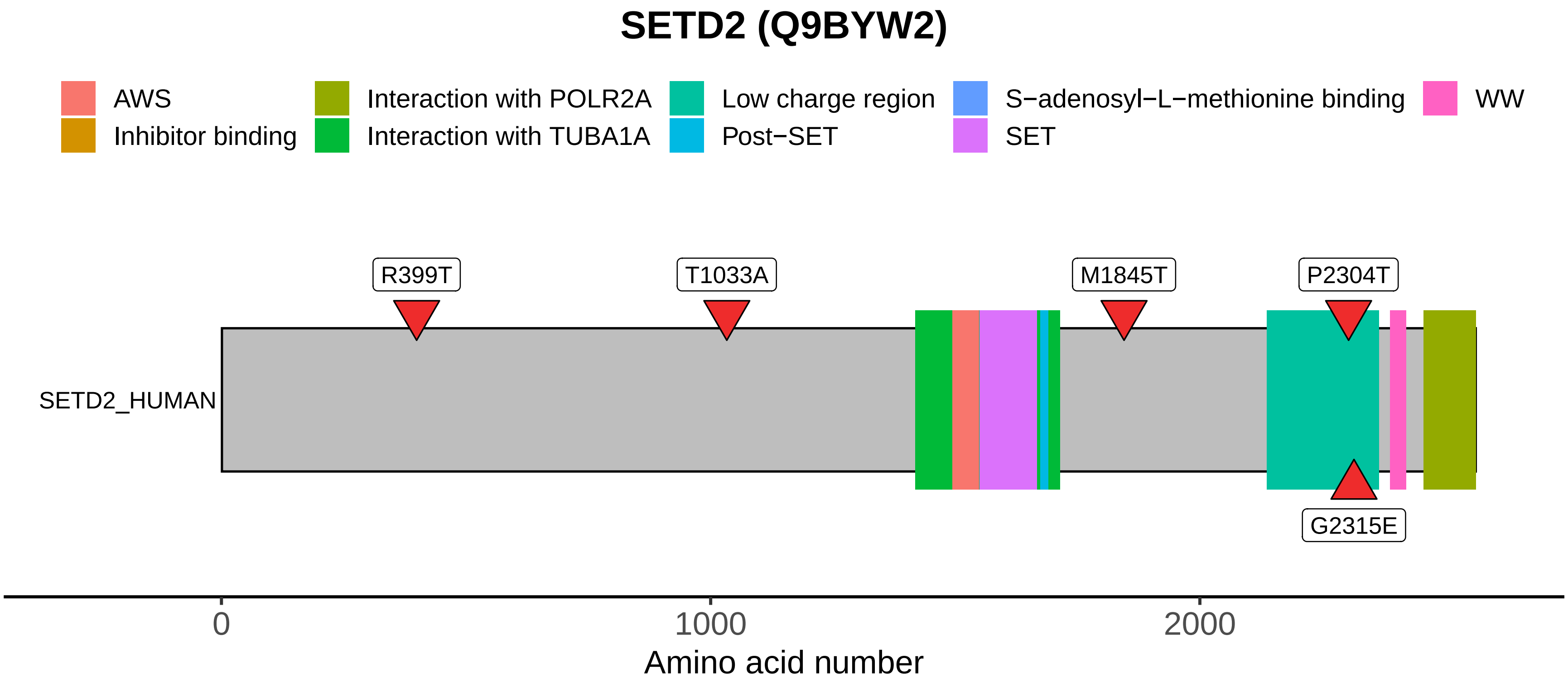
SETD2 (Q9BYW2)
AWS
Inhibitor binding
Interaction with POLR2A
Interaction with TUBA1A
Low charge region
Post−SET
S−adenosyl−L−methionine binding
SET
WW
R399T
T1033A
M1845T
P2304T
G2315E
SETD2_HUMAN
0
1000
2000
Amino acid number

SMC2

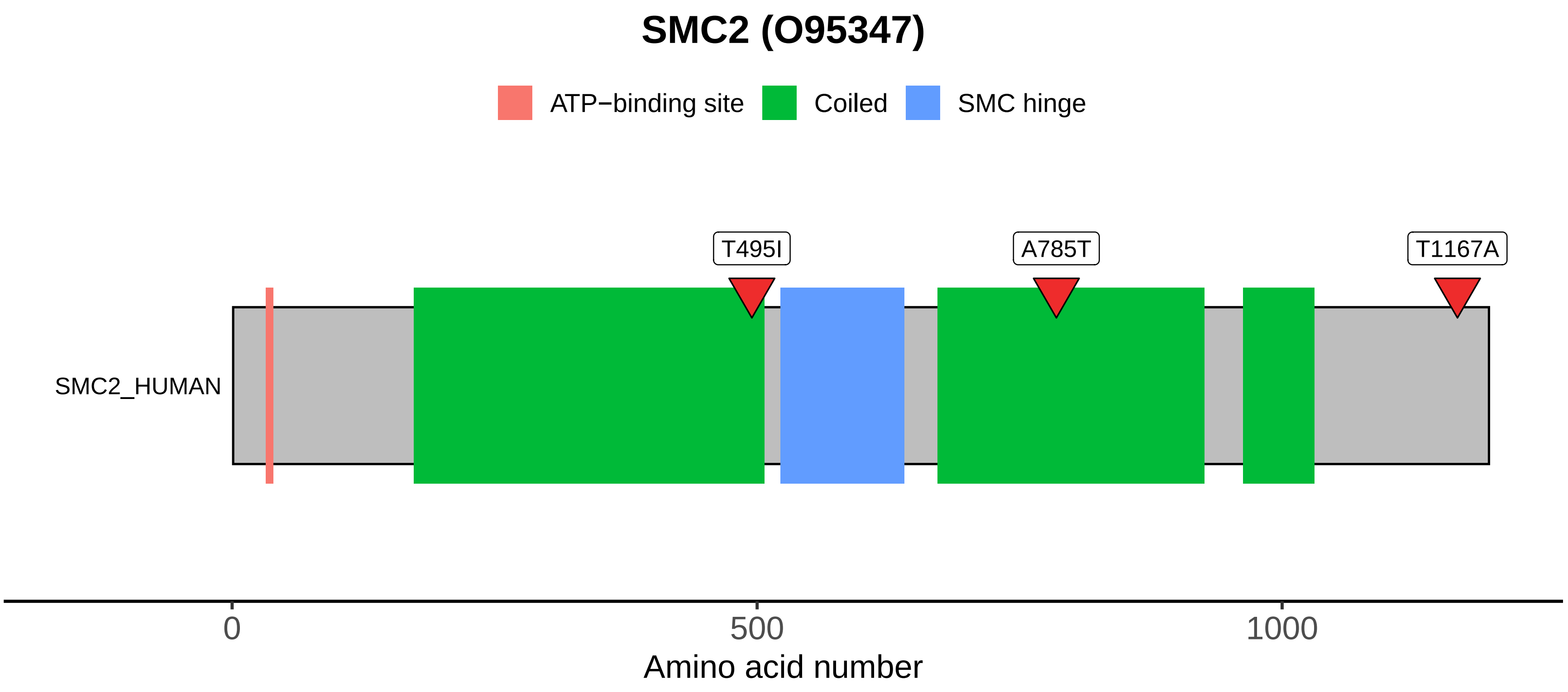

SMC2 (O95347)
ATP−binding site
Coiled
SMC hinge
T495I
A785T
T1167A
SMC2_HUMAN
0
500
1000
Amino acid number

EBF4

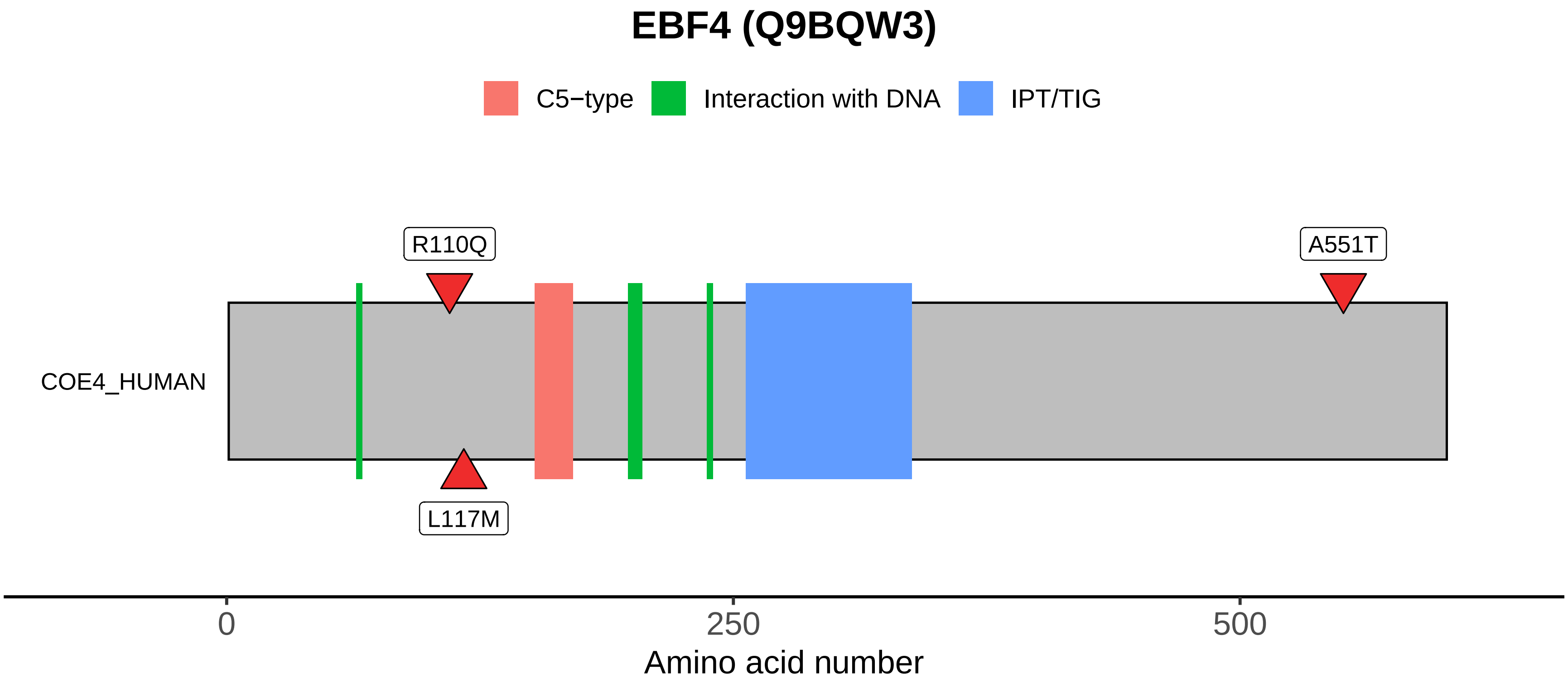

EBF4 (Q9BQW3)
C5−type
Interaction with DNA
IPT/TIG
R110Q
A551T
COE4_HUMAN
L117M
0
250
500
Amino acid number

File S2. Functional organization of the proteins encoded by our 13 candidate susceptibility genes.

Each functional domain or site at the protein level is represented by a colored rectangle. Common structural motifs like beta-transducin repeats, zing-finger and coiled-coils are shown only when they do not belong to an already characterized domain or region. For membrane proteins, information on topology is provided under the form of smaller shaded and labeled rectangles, just above the x-axis. Red arrows identify the position of our candidate variants, labeled with the associated amino acid change. Protein sequences and functional annotations were plotted from the UniProt Knowledgebase (UniProtKB) using R v3.6.1 [1], and the ggplot2 v3.3.2 [2] and drawProteins v1.6 [3] packages. Each protein is labeled by its UniProt stable identifier, provided between brackets in the title after the candidate gene name, and by the recommended protein name, provided at the left of each plot and suffixed with “human”. For PIK3CD, an extra representation suggesting possible implications of the PIK3CD variants on PI3-kinase activity is provided under the form of a ribbon diagram of the p110δ-iSH2 interaction, following [4]. Rare variants near the C-terminal part of the p110δ (encoded by PIK3CD) ABD domain have indeed been functionnally shown to increase PI3-kinase signalling through impaired p85α (encoded by PIK3R1) iSH2 domain-mediated regulation [5]. The protein interaction model was obtained from the Protein Data Bank (https://www.rcsb.org, PDB ID: 5dxu [6]) and processed with PyMOL v2 [7].

Supplemental references

[1] R Core Team (2018) R: A Language and Environment for Statistical Computing. R Foundation for Statistical Computing, Vienna.

[2] Brennan, P. (2018). drawProteins: a Bioconductor/R package for reproducible and programmatic generation of protein schematics. F1000Research, 7.

[3] Wickham H. (2016) ggplot2: Elegant Graphics for Data Analysis. Springer-Verlag, New York.

[4] Shi, H., Hugo, W., Kong, X., Hong, A., Koya, R. C., Moriceau, G., ... & Lo, R. S. (2014). Acquired resistance and clonal evolution in melanoma during BRAF inhibitor therapy. Cancer discovery, 4(1), 80-93.

[5] Heurtier, L., Lamrini, H., Chentout, L., Deau, M. C., Bouafia, A., Rosain, J., ... & Kracker, S. (2017). Mutations in the adaptor-binding domain and associated linker region of p110δ cause Activated PI3K-δ Syndrome 1 (APDS1). Haematologica, 102(7), e278.

[6] Heffron, T. P., Heald, R. A., Ndubaku, C., Wei, B., Augistin, M., Do, S., ... & Olivero, A. G. (2016). The Rational Design of Selective Benzoxazepin Inhibitors of the α-Isoform of Phosphoinositide 3-Kinase Culminating in the Identification of (S)-2-((2-(1-Isopropyl-1 H-1, 2, 4-triazol-5-yl)-5,6-dihydrobenzo[f]imidazo[1,2-d][1,4]oxazepin-9-yl)oxy)propanamide (GDC-0326). Journal of medicinal chemistry, 59(3), 985-1002.

[7] Schrodinger, LLC. (2015). The PyMOL molecular graphics system, v1.8.
